# The mRNA derived MalH sRNA contributes to alternative carbon source utilization by tuning maltoporin expression in E. coli

**DOI:** 10.1080/15476286.2020.1827784

**Published:** 2020-10-12

**Authors:** Ira A. Iosub, Marta Marchioretto, Rob W. van Nues, Stuart McKellar, Gabriella Viero, Sander Granneman

**Affiliations:** a Centre for Synthetic and Systems Biology, University of Edinburgh, Edinburgh0, UK; b Institute of Biophysics, CNR Unit at Trento, Trento, Italy; c Institute of Cell Biology, University of Edinburgh, Edinburgh, UK

**Keywords:** Non-coding RNA, *E. coli*, 3ʹutr, RNA-RNA interactions, stress response, next-generation sequencing

## Abstract

Previous high-throughput studies in Gram-negative bacteria identified a large number of 3ʹUTR fragments that potentially function as sRNAs. Here we extensively characterize the MalH sRNA. We show that MalH is a stable degradation intermediate derived from the 3ʹ end of *malG*, which is part of the maltose uptake operon transcript *malEFG*. Unlike the majority of bacterial sRNAs, MalH is transiently expressed during the transition from the exponential to the stationary growth phase, suggesting that it contributes to adaptation to changes in nutrient availability. Over-expression of MalH reduces expression of general outer membrane porins and MicA, a repressor of the high-affinity maltose/maltodextrin transporter LamB. Disrupting MalH production and function significantly reduces *lamB* accumulation when maltose is the only available carbon source, presumably due to the accumulation of the MicA repressor. We propose that MalH is part of a regulatory network that, during the transition phase, directly or indirectly promotes accumulation of high-affinity maltose transporters in the outer membrane by dampening competing pathways.

## Introduction

Efficient adaptation to changing environmental conditions is fundamental to bacterial survival and success. The environments that bacteria naturally encounter typically lack optimal concentrations of nutrients, and available substrates can be found in complex mixtures. Thus, free-living bacteria often have to switch from catabolising one nutrient to another and co-utilize multiple substrates. Moreover, nutrient availability and bacterial metabolic strategies are closely linked to virulence [1–4]. Understanding bacterial physiology from a systems point of view requires a thorough understanding of how adaptation to changes in nutrient composition in the environment is controlled.

The most common experimental approach used to study changes in nutrient adaptation is using LB (Lysogeny broth) as a growth medium. Although not fully replicating natural environments, LB batch cultures have been widely used to model bacterial growth and provide a suitable system for studying bacterial growth under nutrient-limited conditions. LB is particularly well suited for investigating glucose-limited growth because glucose is only found in trace amounts in this medium [5]. Initially, *Escherichia coli* utilizes carbon sources from LB in a sequential manner [6,7]. First, it consumes its preferred nutrient, glucose. When the concentration of glucose drops below levels that allow sustained population growth, *E.coli* can transition to a scavenging mode [8], utilizing multiple alternative carbon sources from LB’s carbohydrate mix, including maltodextrins, D-mannose, melibiose, D-galactose, and ribose [5]. These carbon sources are not highly abundant in LB; thus by the time growth reaches the stationary phase, the population relies on the highly abundant amino acids and peptides [7,9]. The growth on sub-saturating nutrient levels and the changes that occur during the transition from exponential to stationary growth phase requires the expression of the appropriate transport systems for nutrient import, metabolic enzymes, as well as additional changes in gene expression for maintaining cellular homoeostasis. Given their general role in nutrient uptake, outer membrane proteins (OMPs) expression is coordinated with changes in nutrient availability [8,10]. Moreover, cells must tightly balance the expression of general and specialized transporters in the outer membrane to maintain membrane integrity [11].

While transcription factors are considered to have the main contribution in coordinating these changes in gene expression, post-transcriptional regulators, including Hfq and its associated base-pairing sRNAs, provide a key additional layer of regulation by controlling target mRNA transcription termination, degradation and/or translation [12]. Some sRNAs have direct roles in controlling nutrient uptake metabolism and transporter expression. For example, SdhX is involved in acetate metabolism [13,14], SgrS downregulates the expression of a major glucose transporter [15], Spot42 regulates catabolite repression [16], GcvB regulates peptide and amino acid transporters [17–19] and MicA represses the expression of maltoporin (LamB), the maltose-specific porin [20,21]. Additionally, MicA and RybB downregulate the expression of several OMPs [21–24].

A rapidly emerging class of sRNAs are encoded within mRNA genomic loci rather than at independent sites in the genome. Indeed, the 3ʹUTR fragments of many mRNAs co-immunoprecipitate with Hfq [25,26] and a subset have been shown to be functional Hfq-dependent sRNAs. While some mRNA-derived sRNAs are transcribed from regions overlapping a 3ʹUTR (e.g. DapZ [26]), others originate by the cleavage of a parental mRNA by RNases [13,14,27–29] or are independently transcribed before they are processed by RNases [30]. RNase E has been shown to be involved in the biogenesis of certain mRNA-cleaved sRNAs, but for others, the RNase responsible for processing has yet to be identified [30]. Moreover, 3ʹUTR-derived sRNAs can also associate with the RNA chaperone ProQ (e.g. RaiZ [28,31,32]). The recovery of hybrid fragments involving 3ʹUTRs of mRNAs in Hfq CLASH and RIL-seq data expanded the interactomes of known 3ʹUTR-derived sRNAs, but also uncovered many new 3ʹUTR-derived sRNA candidates, highlighting the prevalence of this class of riboregulators [33,34]. A few mRNA-derived sRNAs have been characterized in detail and have been shown to regulate critical aspects of bacterial physiology, ranging from the regulation of membrane assembly and composition [27,30,35,36], to controlling nitrate [29], acetate [13] and amino acid metabolism [37]. Their pervasiveness and key roles underscore the importance of 3ʹUTRs as reservoirs of sRNAs in enterobacteria.

Despite playing a fundamental role in rewiring gene expression, the functions of most Hfq-associated sRNAs during the transition from exponential to stationary phase have remained unexplored. To fill this gap, we recently employed CLASH to map the sRNA interactomes mediated by Hfq in *E.coli* during the exponential, transition and stationary phases of growth [33]. Our interaction maps have expanded the regulatory networks [34] with hundreds of novel sRNA–mRNA interactions, many of them specific to the transition stage. Here, we focussed our analyses on interactions that were specifically recovered during this phase. We identified and characterized a 3ʹUTR-derived sRNA, which we refer to as MalH. Unlike the majority of bacterial sRNAs, MalH is transiently expressed during the transition phase. We demonstrate that MalH is a degradation intermediate of the 3ʹ end of the *malG* transcripts, the last transcript of the *malEFG* polycistron encoding components of the maltose transport system [38]. We show that over-expression of MalH downregulates several mRNAs encoding major OMPs and directly or indirectly reduces the levels of MicA, an sRNA that downregulates the high-affinity maltose transporter LamB [20,21]. In addition, mutations in the seed sequence of the endogenous MalH reduce the accumulation of the *lamB* mRNA, presumptively caused by MicA de-repression.

We propose that the MalH sRNA is part of a regulatory network that, during the transition phase, promotes accumulation of high-affinity maltose transporters in the outer membrane by dampening competing pathways.

## Materials and methods

### Bacterial strains and culture conditions

An overview of the bacterial strains used in this study is provided in Supplementary Table 2. The *E. coli* MG1655 and TOP10 strains served as parental strains. Cells were grown in Lysogeny Broth (LB) or minimal medium with supplements (1xM9 salts, 2 mM MgSO_4_,0.1 mM CaCl_2_, 0.03 mM thiamine, 0.2% carbon source) at 37°C under aerobic conditions with shaking at 200 rpm. The media were supplemented with antibiotics where required at the following concentrations: ampicillin (Sigma, UK, A9518) – 100 µg/ml, chloramphenicol (Corning, – S, C239RI) – 25 µg/ml, kanamycin (Gibco, US–11,815-024) – 50 µg/ml. Where indicated, 0.2% glucose or maltose were used. For induction of sRNA expression from plasmids 0.2% L-arabinose (Sigma, A3256) was used. An overview of plasmids used in this study is provided in Supplementary Table 3.

### Construction of sRNA expression plasmids

For the sRNA pulse over-expression constructs (Supplementary Table 3), the sRNA gene of interest was cloned at the transcriptional +1 site under P*lacOara* control by amplifying pBAD+1 plasmid [25] by inverse PCR using Q5 DNA Polymerase (NEB). The pBAD+1 template is derived from pBAD*myc*HisA. The sRNA genes and seed mutants (SM) were synthesized as ultramers (IDT, Belgium), which served as the forward primers (Supplementary Table 4). The reverse primer (oligo pBAD+1_5P_rev) bears a monophosphorylated 5ʹ-end. The template plasmid was digested with 10 U DpnI (NEB) for 1 h at 37°C and the PCR product purified by ethanol precipitation. The pBAD-sRNA linear PCR product was circularized by self-ligation and transformed in DH5α competent cells. Positive transformants were screened by Sanger sequencing (Edinburgh Genomics, Edinburgh, UK). The control plasmid pBAD+1 was constructed similarly by self-ligation of the PCR product generated from oligonucleotides pBAD+1_XbaI_fwd and pBAD+1_5P_rev.

### Construction of mRNA-superfolder GFP fusions

To generate constitutively expressed mRNA-*sfGFP* fusions for the fluorescence reporter studies, the 5ʹUTR, start codon and first ~5 codons of target genes were cloned under the control of PLtetO-1 promoter in a pXG10-SF backbone as previously described [19,39]. Derivatives of the target–GFP fusion plasmids harbouring seed mutations (SM) were generated using synthetic-mutated gene-fragments (Supplementary Table 4) (IDT, Belgium). To prepare the inserts, the target region of the mRNA of interest was either amplified by PCR from *E. coli* genomic DNA or synthesized as g-blocks (IDT, Belgium) and cloned using NheI and NsiI restriction sites and transformed in TOP10 cells. Transformants were screened by restriction digest analysis and verified by Sanger sequencing (Edinburgh Genomics, Edinburgh, UK).

### GFP reporter system to quantify sRNA effect on target expression

A two-plasmid system was used to express each sRNA, and mRNA-*sfGFP* fusions [19,39] with modifications. The sRNA and sfGFP-fusion plasmids were co-transformed in *E. coli* TOP10 cells by electroporation and cells were maintained on dual selection with ampicillin and chloramphenicol. In TOP10 cells, the mRNA-*sfGFP* constructs are constitutively expressed, whereas the sRNA expression requires L-arabinose induction. TOP10 cells were chosen for this experiment as they are deficient in RecA recombinase, which reduces intermolecular recombination between plasmids. The expression of sfGFP-fused targets in the presence or absence of sRNAs was quantified at the protein level, by plate reader experiments and at the RNA level, by RT-qPCR (see below).

For the plate reader experiments, a single colony of the bacterial strain harbouring an sRNA-target-sfGFP combination was inoculated in a 96-well Flat Bottom Transparent Polystyrene plate with lid (Thermo Scientific, 152,038) and cultured overnight at 37°C in 100 μl LB supplemented with antibiotics and L-arabinose (Sigma, A3256) to induce expression of sRNAs. Next day, each overnight inoculum was diluted 1:100 by serial dilution, in triplicate, in LB with freshly prepared L-arabinose to a final volume of 100 μl. Cultures were grown in a 96-well plate in an Infinite 200 Pro plate reader (Tecan) controlled by i-control software (Tecan) for 192 cycles at 37°C with 1 min orbital shaking (4 mm amplitude) every fifth minute. To monitor the optical density over time, the following parameters were used: wavelength 600 nm, bandwidth 9 nm. Fluorescence was monitored with excitation wavelength 480 nm, bandwidth 9 nm and emission wavelength 520 nm, bandwidth 20 nm. Measurements were recorded at 5-min intervals, by top reading. Raw data were processed following guidance from previous reports [19]. First, the range of linearity of increase of fluorescence with OD_600_ was identified for all individual triplicates. Only the linearity range common to all triplicates was considered for further analysis. For each set of triplicates, the mean fluorescence was calculated at each OD_600_. To correct for background and cell autofluorescence, the mean fluorescence of a strain with plasmid pXG-0 was subtracted from all strains with GFP plasmids at the equivalent OD_600_. Ultimately, a curve was generated for each sample, plotting the background-corrected fluorescence (GFP) versus OD_600_. The experiments were performed for three biological replicates, and mean values and standard error of the means calculated for each strain.

### RT-qPCR

For the superfolder GFP expression experiments, total RNA (12.5 µg) was treated with 2 U of Turbo DNase (Thermo Scientific, AM2238) for 1 hour at 37°C in a 10 μl reaction in the presence of 2 U SuperaseIn RNase inhibitor (Thermo Scientific, AM2694). The DNase was inactivated by 10 minutes incubation at 75°C. Reverse transcription (RT) was performed in a single reaction for all target genes of interest using a mix of gene-specific RT primers at 3.5 μM concentration each. After the addition of 2.5 μl RT primer mix, the RNA and primers were denatured at 70°C for 3 min, then snap chilled and incubated on ice for 5 min. RT was performed for 1 h at 55°C with SuperScript III (Thermo Scientific, 18,080,051) using 5 μl of RNA-RT primers mix in 10 μl final volume (100 U Superscript III, 2.5 mM DTT, 1xFS Buffer, 0.75 mM dNTPs) in the presence of 1 U RNasin (Promega, N2115). RT was followed by treatment with 5 U RNase H for 30 min at 37°C to remove the RNA from the RNA-cDNA duplexes. The cDNA was diluted 10-fold with DEPC water. Quantitative PCR was performed on 50 ng of DNAse I-treated total RNA using the Brilliant III UltraFast SYBR Green QPCR Master Mix (Agilent, #600,883). For sRNA pulse-over-expression and sRNA deletion experiments, the Luna Universal One-Step RT-qPCR Kit (NEB, E3005E) was used according to the manufacturer’s instructions. The qPCRs were run on a LightCycler 480 (Roche), and the specificity of the product was assessed by generating melting curves, as follows: 65°C-60s, 95°C (0.11 ramp rate with 5 acquisitions per °C, continuous). The data analyses were performed with the lightcycler software, at default settings: Absolute Quantification/Fit Points for Cp determination and Melt Curve Genotyping. The qPCR for all samples was performed in technical triplicate. Outliers from the samples with technical triplicate standard deviations of Cp >0.3 were discarded from the analyses. To calculate the fold-change relative to the control, the 2^−ddCp^ method was employed, using *recA* or 5S rRNA (*rrfD*) as the reference genes where indicated. Experiments were performed for minimum of two biological replicates, and the mean fold-change and standard error of the mean were computed. Unless otherwise stated, the significance of the fold-change difference compared to the reference sample control (for which fold-change = 1 by definition) was tested with a one-sample t-test. Oligonucleotides used for RT-qPCR are provided in Supplementary Table 4.

### MalH over-expression studies

For pulse over-expression studies overnight MG1655 cultures containing pBAD::sRNA and empty pBAD+1 control plasmids were inoculated in fresh LB-ampicillin medium at a starting OD_600_ of 0.05 and grown aerobically at 37°C to OD_600_ 0.4. Pre-induction (0 min) and post-induction samples were harvested. For induction, cultures were supplemented with L-arabinose (Sigma, A3256) and rapidly collected by filtration and flash-frozen in liquid nitrogen at the indicated time-points. RNA was extracted from samples harvested 15 minutes post-induction (OD_600_ between 0.6 and 0.7) three biological replicate time-series, followed by RNA-seq library preparation, next-generation sequencing and DESeq2 [40] analysis of differentially expressed genes (Supplementary Table 1).

### RNA extraction

RNA extraction was performed using the guanidium thiocyanate (GTC) method as previously described [41,42]. Briefly, 5 ml of cell culture was harvested at exponential phase by centrifugation. Cell pellets were resuspended in 100 µl GTC-Phenol (pH 7; 1:1 ratio) and lysed by vortexing the cells for 5 min with 100 µl of Zirconia beads (0.1 mm; Biospec products 11079101z). Subsequently, 550 µl of GTC was added and the mixture was vortexed for several minutes and incubated at 65ºC for 10 minutes and then 10 minutes on ice. Phases were separated by adding 300 µl chloroform isoamylalcohol (24:1) and 1/10^th^ of a volume of 3 M NaAc (pH 5.2) followed by vigorous vortexing. After 5 minutes of centrifugation in an Eppendorf centrifuge (14 k rpm), the RNA was purified from the upper phase via two additional rounds of phenol-chloroform extraction and precipitated with 3 volumes of 96% cold ethanol.

### RNA-seq

For the over-expression analysis of MalH, we generated RNA-seq libraries using an in-house protocol. Genomic DNA was removed by incubating 10 μg of total RNA with 2 U Turbo DNase (Thermo Scientific, AM2238) in a 50 μl final volume for 30 minutes at 37°C in the presence of 10 U SuperaseIn RNase Inhibitor (Thermo Scientific, AM2694). RNA was subsequently phenol-chloroform extracted and purified by ethanol-precipitation. Ribosomal RNA was removed with the Ribo-Zero rRNA Removal Kit (Gram-Negative Bacteria; Illumina, MRZGN126) according to the manufacturer’s instructions. Successful rRNA depletion was verified on the Agilent 2100 Bioanalyzer. The RNA was fragmented for 5 min at 95°C in the presence of Superscript III buffer (Invitrogen) followed by a five-minute incubation on ice. Reverse-transcription (RT) was performed with Superscript III (Thermo Scientific, 18,080,044) in 20 μl reactions according to manufacturer’s procedures using 250 ng of ribosomal RNA depleted RNA and 2.5 μM random hexamers (PE_solexa_hexamer, oligo 73, Supplementary Table 4). The RNA and free primers were degraded using 20 U of Exonuclease I (NEB, M0293l) and 50 U RNaseIf (NEB, M0243S) and the cDNA was purified with the DNA Clean & Concentrator 5 kit (Zymo Research). Ligation of the 5ʹ adapter (P5_phospho_adapter, oligo 39) to the cDNA was performed using CircLigase II (Lucigen, CL9021K) for 6 hours at 60°C, followed by a 10-min inactivation at 80°C. The cDNA was purified with the DNA Clean & Concentrator 5 kit. Half of the cDNA library was PCR amplified using Pfu polymerase (Promega, M7745) using the P5 forward PCR oligonucleotide and barcoded BC reverse oligonucleotides (200 nM; Supplementary Table 4; 95°C for 2 min, 95°C for 20s, 52°C for 30 s and 72°C for 1 min, and a final extension of 72°C for 5 min. 20 cycles of amplification). The PCR products were treated with Exonuclease 1 (NEB, M0293L) for 1 h at 37°C and purified by ethanol precipitation. Libraries were resolved on a 2% MetaPhor agarose gel 200–500 bp fragments were gel-extracted using the MinElute kit. All libraries were quantified on a 2100 Bioanalyzer using the High-Sensitivity DNA assay (Agilent, 5067–4627). Individual libraries were pooled in equimolar amounts. Paired-end sequencing (75 bp) was performed by Edinburgh Genomics on the Illumina HiSeq 4000 platform.

### Seed-mutant studies

Wild-type MG1655 and seed-mutant strains were grown overnight in minimal medium with glucose. Next day, each starter culture was split and inoculated at OD_600_ 0.05 in fresh M9 medium with glucose or maltose as the sole carbon source. Growth was monitored and cells were harvested at OD_600_ 0.5. Total RNA was extracted, and gene expression was quantified by RT-qPCR or Northern Blot.

### Polysome profiling analyses

Wild-type *E. coli* MG1655 containing empty pBAD plasmid and an isogenic strain containing pBAD: MalH was grown in LB until OD_600_ 0.4, then treated for 15 minutes with L-arabinose (Sigma, A3256) to induce over-expression of MalH, and cycloheximide (Sigma, C4859-1 ML) at a final concentration of 100 µg/ml for 3 minutes. 200 ml of cells (OD_600_ 0.6–0.7) were harvested by rapid filtration and flash frozen. The cells were washed in ice-cold PBS supplemented with 100 µg/ml cycloheximide.

Polysomal profiling was performed according to previously described protocols [43,44] with minor changes in the lysis buffer (10 mM NaCl, 10 mM MgCl_2_, 10 mM Tris-HCl (pH 7.5), 100 µg/ml cycloheximide, 1% (w/v) Na-Deoxycholate, 1 U RQ DNAse I (Promega, M6101), 0.6 U/mL RiboLock (Thermo Scientific, EO0381) in DEPC water). Lysates were kept on ice for 30 min, centrifuged 3X at 15,000 g for 10 min. The supernatants were loaded on a linear 10%–30% [w/v] sucrose gradient and centrifuged for 4 hours using an SW41 rotor at 40,000 rpm in a Beckman Optima XPN-100 Ultracentrifuge. Fractions of 1 mL in volume were collected monitoring the absorbance at 254 nm with the UA-6 UV/VIS detector (Teledyne Isco). Fractions from the entire gradient (total RNA) and from the fractions corresponding to ribosomes (70S) or polysomes (polysomal RNA) were pooled and RNA was purified by acid phenol–chloroform extraction as previously described [45].

### Terminator™ 5′-PhosphateDependent Exonuclease treatment

Ten micrograms of total RNA extracted from cell-samples at OD_600_ 1.2 and 1.8 were treated with 5ʹ-Terminator Dependent Exonuclease (Lucigen, TER51020) as per manufacturer instructions using Buffer A. The reaction was terminated by phenol extraction and ethanol precipitation, and the RNA was loaded on 8% polyacrylamide-urea gels and transferred to nylon membranes that were probed for MalH, CpxQ, RybB and 5S rRNA (Supplementary Table 4).

### Primer extension analysis

One microgram total RNA was reverse-transcribed using SuperScript III reverse transcriptase (Thermo Scientific, 18,080,051) using ^32^P -radiolabelled oligonucleotides as primers (Supplementary Table 4). Primers were added to the RNA and annealing was performed by heating the samples at 85°C for 3 min and then snap chilling them on ice. The RT was performed for 1 h at 45°C, followed by Exonuclease I and RNaseIf (NEB M0293L and M0243S) (0.5 μl each) treatment for 30 minutes at 37°C. Reactions were stopped by mixing with an equal volume of 2XRNA loading dye (NEB, B0363S), 2 minute incubation at 95°C and snap chilled. The sequencing ladders were prepared with Sequenase v2.0 (Thermo Scientific, 70775Y200UN) according to specified instructions. Samples were resolved on 6% PAA/8 M TBE-urea gels and visualized using the FujiFilm FLA5100 scanner.

### Construction of the MalH seed-mutant strain

To mutate the chromosomal copy of MalH, we used the λRed system [46]. We amplified the integration cassette from plasmid pKD4 with ultramers 895 and 896, containing homology regions to the coding sequence of *malG*, the desired MalH sequence and to the region immediately downstream of the Rho-independent terminator, respectively. With this design, the scar after removal of the *Kan*^r^ cassette was expected at a site outside the MalH/*malG* sequence. The PCR product was electroporated in *E. coli* MG1566 strains carrying the pKD46 plasmid from which λRed recombinase was induced with 10 mM L-arabinose. Correct replacement of the MalH seed sequence was screened by colony PCR using primer pairs: 725 & 909 and 726 & 910 (Supplementary Table 4). The antibiotic resistance cassette was removed from substitution mutants by FLP-recombinase expressed constitutively from pE-FLP [47]. Successful allele replacement was confirmed by Sanger sequencing.

### Northern blot analysis

Total RNA was extracted from cell lysates by GTC-Phenol extraction. For large RNA fragments, 10 μg of total RNA was resolved on a 1.25% BPTE-gel (pH 7) and transferred to a nylon membrane (HyBond N+, GEHealthcare, RPN1210B) by capillarity. For short RNA fragments, 10 μg total RNA was separated on an 8% polyacrylamide TBE-Urea gel and transferred to a nitrocellulose membrane by electroblotting for 4 h at 50 V. Membranes were pre-hybridized in 10 ml of UltraHyb Oligo Hyb (Thermo Scientific, AM8663) for 1 h and probed with ^32^P-labelled DNA oligo at 42°C for 12–18 hours in a hybridization oven. The sequences of the probes used for Northern blot detection are detailed in Supplementary Table 4. Membranes were washed twice with 2xSSC +0.5% SDS solution for 10 minutes and visualized using a Phosphor imaging screen and FujiFilm FLA-5100 Scanner (IP-S mode). For detection of highly abundant species (5S rRNA), autoradiography was used for exposure.

### Computational analysis

#### Pre-processing of the raw sequencing data

Raw sequencing reads in fastq files were processed using a pipeline developed by Sander Granneman, which uses tools from the pyCRAC package [48]. The entire pipeline is available at https://bitbucket.org/sgrann/. The CRAC_pipeline_PE.py pipeline first demultiplexes the data using pyBarcodeFilter.py and the in-read barcode sequences found in the L5 5ʹ adapters. Flexbar then trims the reads to remove 3ʹ-adapter sequences and poor-quality nucleotides (Phred score <23). Using the random nucleotide information present in the L5 5ʹ-adaptor sequences, the reads are then collapsed to remove potential PCR duplicates. The reads were then mapped to the *E. coli* MG1655 genome using Novoalign (www.novocraft.com). To determine to which genes the reads mapped to, we generated an annotation file in the Gene Transfer Format (GTF). This file contains the start and end positions of each gene on the chromosome as well as what genomic features (i.e. sRNA, protein-coding, tRNA) it belongs to. To generate this file, we used the Rockhopper software [49] on *E. coli* rRNA-depleted total RNA-seq data (generated by Christel Sirocchi), a minimal GTF file obtained from ENSEMBL (without UTR information). The resulting GTF file contained information not only on the coding sequences but also complete 5ʹ and 3ʹ UTR coordinates. We then used pyReadCounters.py with Novoalign output files as input and the GTF annotation file to count the total number of unique cDNAs that mapped to each gene.

*Differential expression analyses*.

DESeq2 [40] was used on raw read counts generated by pyReadCounters from the pyCRAC package [48] to detect differentially expressed genes. Three MalH pulse-over-expression datasets were compared to three pBAD Control over-expression datasets. Only differentially expressed genes that had an adjusted p-value of 0.05 or lower were considered significant.

#### Normalization steps

To normalize the read count data generated with pyReadCounters.py and to correct for differences in library depth between time-points, we calculated Transcripts Per Million reads (TPM) for each gene. Briefly, for each time-point the raw counts for each gene were first divided by the gene length and then divided by the sum of all the values for the genes in that time-point to normalize for differences in library depth. The TPM values for each OD_600_ studied were then log_2_-normalized.

#### Multiple sequence alignments and conservation analyses

The homologous sequences of MalH in other enterobacteria were retrieved by BLAST. JalView was used for the multiple sequence alignments, using the MAFFT algorithm [50].

### Data and Code availability

The MalH over-expression RNA sequencing data have been deposited on the NCBI Gene Expression Omnibus (GEO) with accession number GSE149059. The RNA-seq data generated from cells harvested at the various growth stages are available from NCBI GEO with accession number GSE123048. The python pyCRAC [48], kinetic-CRAC and GenomeBrowser software packages used for analysing the data are available from https://bitbucket.org/sgrann (pyCRAC up to version 1.4.3), https://git.ecdf.ed.ac.uk/sgrannem/, https://pypi.org/project/pyCRAC and https://pypi.org/project/GenomeBrowser.

## Results

**Figure 1.**
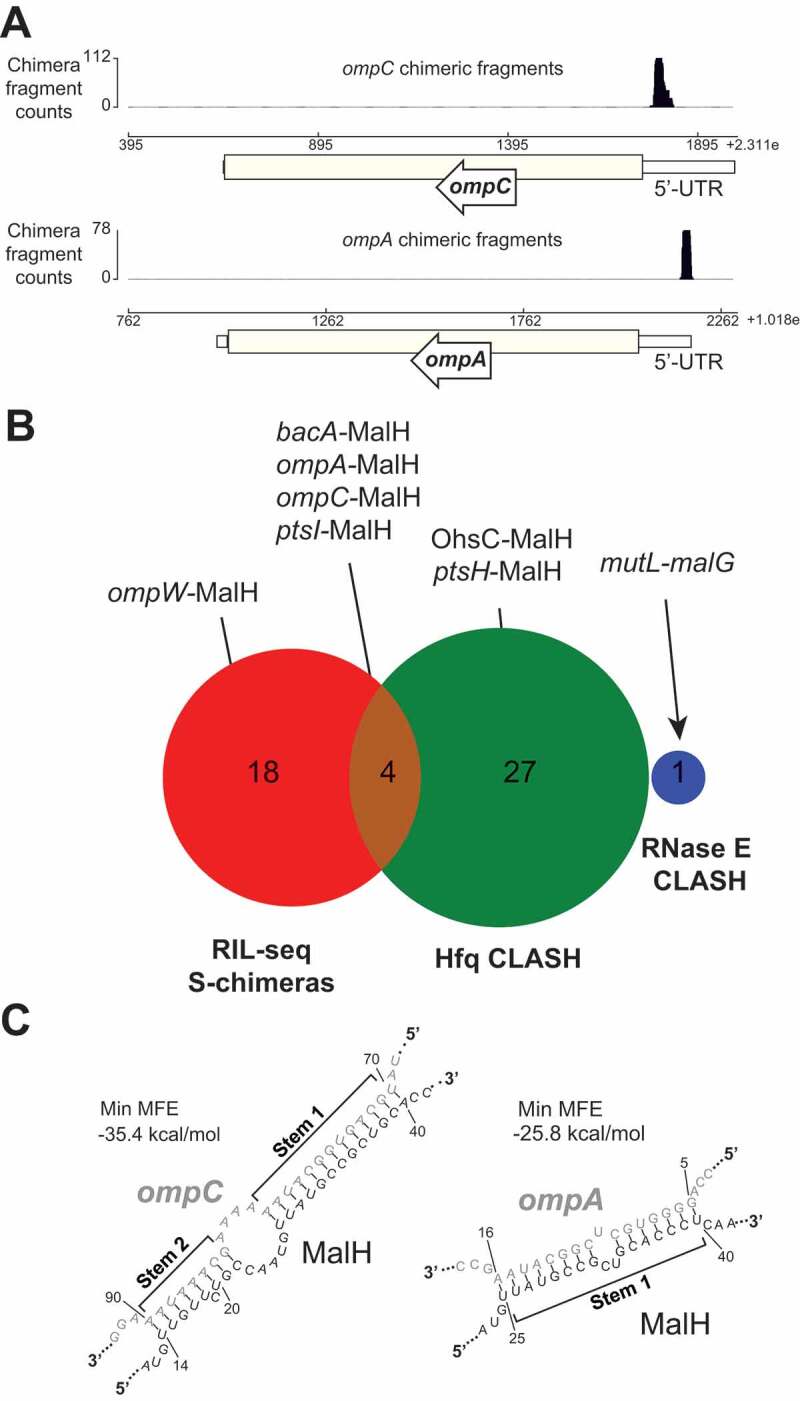
MalH is a 3ʹUTR-derived sRNA that base-pairs with major porin mRNAs

To characterize MalH, we analysed published *E. coli* RNA–RNA interaction data [33,34]. This revealed that MalH could potentially base-pair with 5ʹUTRs of mRNAs encoding several major porins, including the highly abundant *ompC* and *ompA* mRNAs (Fig. 1A-C). An interaction between MalH and another outer membrane porin (*ompW*) was also detected in the RIL-seq data [34], implying that MalH could potentially regulate many outer membrane porins. The most abundant interactions of MalH with mRNAs in the published datasets (*ompC* and *ompA*) utilized mostly one region in the *malG* 3ʹUTR for base-pairing (Fig. 1C, Stem 1). This suggests that the corresponding site on the predicted sRNA is likely the main seed. The predicted interaction between MalH and *ompC* is unusually long and consists of two stems interrupted by a bulge (Fig. 1C), suggesting that these two RNAs form a stable complex. Conservation analyses and *in silico* target predictions (CopraRNA [51,52]) indicated that the seed sequence part of stem 1 is relatively well conserved (Supplementary Fig. 1A) and could be utilized for the regulation of multiple mRNA targets (Supplementary Fig. 1B). The sequences in MalH and *ompC* that form stem 2 (Fig. 1C) are less well conserved (Supplementary Fig. 1A and 2A).

(A) The three genome browser tracks show the distribution of mRNA and sRNA fragments (*ompC* and *ompA*) that were fused to MalH fragments in chimeric reads. (B) MalH-base-pairing interactions found in Hfq CLASH, RIL-seq S-chimera data (log and stationary phase) and RNase E CLASH data. The number of interactions found in the individual data sets or in both are shown (C) MalH forms stable duplexes with the 5ʹUTR of porin-encoding mRNAs. *In silico* prediction (RNADuplex [53]) of hybrid structures derived from the most abundant MalH chimeric reads with the indicated transcripts. The min. MFE is the minimum folding energy assigned by RNAcofold.

**Figure 2.**
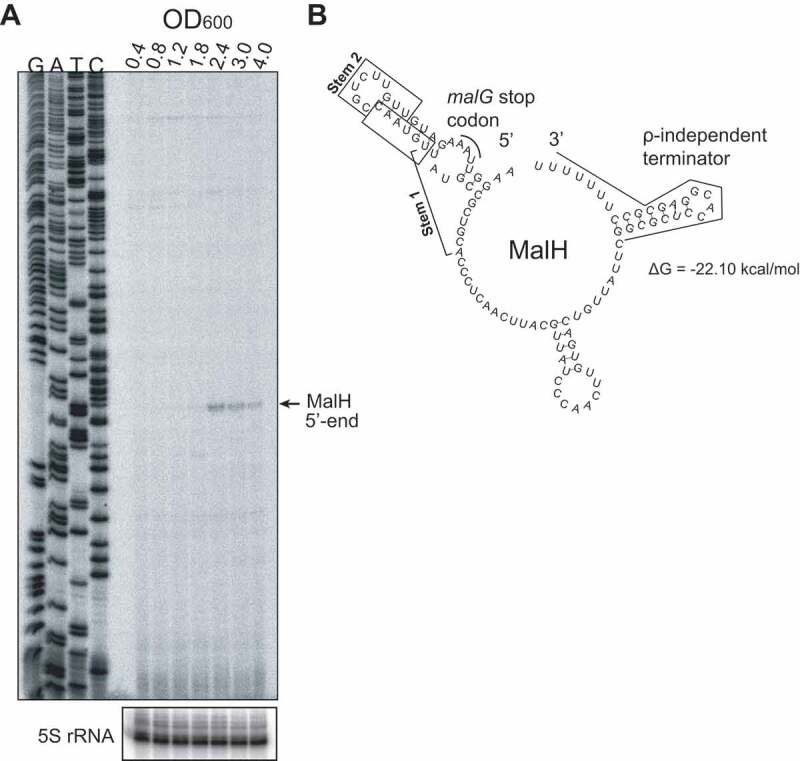
MalH is a 104nt sRNA

**Figure 3.**
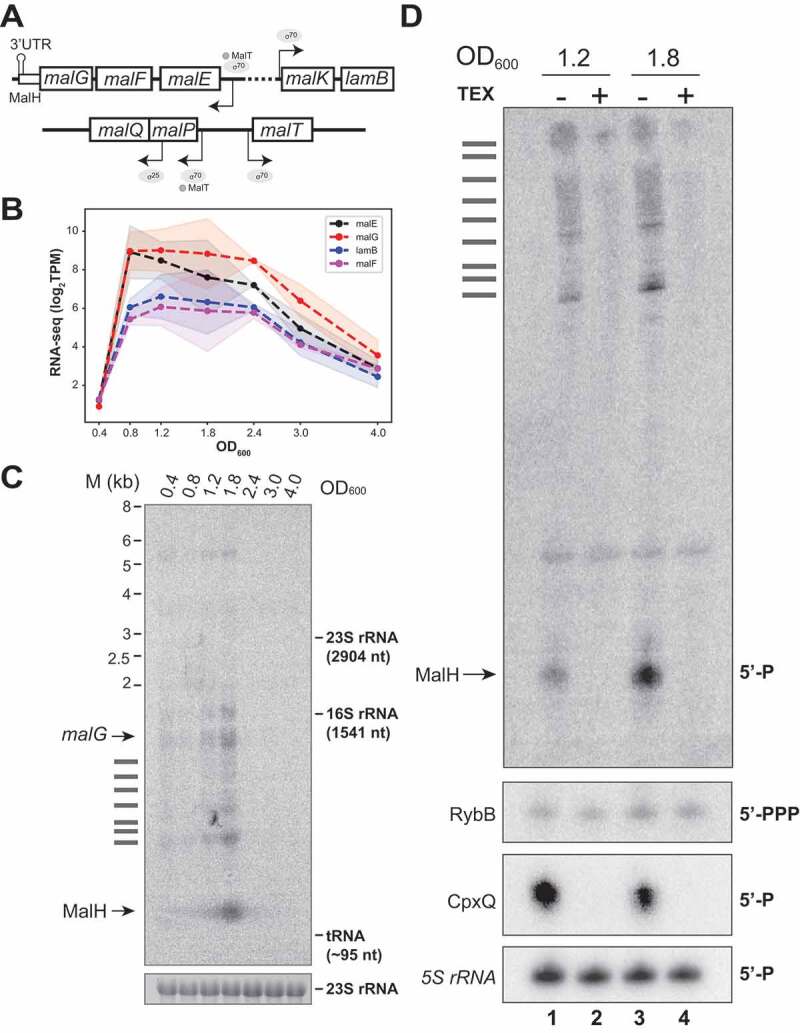
MalH is a degradation product that emerges at the transition between exponential and stationary phases of growth

To obtain the full-length sequence of MalH, we performed primer extension analyses to map the 5ʹ end of the sRNA (Fig. 2A). This revealed that MalH is a 104 nt sRNA that contains part of the *malG* coding sequence, the stop codon and the Rho-independent terminator (Fig. 2B). Next, we analysed the expression of this operon using our previously published RNA-seq data that contained gene expression information at various different growth stages in LB medium [33] (see Fig. 1B of this study for LB growth curves). The expression of *malEFG*, similarly to all other transporters and enzymes of the maltose system, is under the control of the MalT transcription factor that is bound to the maltotriose inducer and dependent on CRP-cAMP (Fig. 3A). Thus, these genes are expressed in the presence of maltose or maltodextrins and repressed in the presence of glucose due to catabolite repression. In LB, the steady-state levels of *malG* recapitulate a similar transient expression profile peaking between OD_600_ 1.2 and OD_600_ 2.4 (Fig. 3B).

(A) Primer extension analysis to map the 5ʹ-terminus of MalH, using *E.coli* total RNA extracted from cells growing at different optical densities (OD_600_ 0.4–4.0) and an RT oligo that anneals to the 3ʹ-end region of the sRNA; G, A, T, C lanes represent the sequencing ladder; 5S rRNA probes were used to generate a loading control. (B) *In silico* prediction of full-length MalH secondary structure (RNAfold [53]); the *malG* stop codon and the Rho-independent terminator are highlighted with red and black, respectively; the minimum free energy of folding is noted below the structure.

(A) Schematic representation of the *mal* regulon gene structure. (B) The *mal* regulon gene expression peaks at the transition between exponential and stationary phases of growth: the plot shows averages (dotted line) and standard deviations (shaded regions) of log_2_(TPM) normalized RNA steady-state levels from four different transcripts that encode proteins involved in maltose uptake (y-axis); the x-axis indicates the cell densities (OD_600_) at which samples were taken. (C) Northern blot using total RNA from *E. coli* harvested at different cell densities (OD_600_) probed with an oligo antisense to *malG* 3ʹUTR; ‘M’ indicates the size ladder. The 23S rRNA was used as the loading control; the identity of the bands is indicated at the left of the panel; horizontal bars indicate *malEFG* degradation intermediates. (D) MalH is a degradation product: Northern blot using total RNA from cells at indicated OD_600_ with (+; lanes 2 and 4) or without (-; lanes 1 and 3) 5ʹ-Phosphate-Dependent Exonuclease (TEX) treatment. The sRNAs RybB (5ʹ-PPP) and CpxQ (5ʹ-P) were used as negative and positive controls, respectively. The 5S rRNA was used as a loading control. The text on the right of the blot indicates the phosphorylation state of the 5ʹ-termini for each sRNA. Note that the 5S rRNA has a 5ʹ-P but is not degraded by the TEX enzyme.

MalH sRNA levels generally peaked around OD_600_ 1.8–2.4 (Fig. 2A and Fig. 3C). We suspect that this variability in MalH expression is likely due to experimental noise as a result of slight differences in sugar content of the various batches of LB used during the project. Nevertheless, we consistently observed a transient induction of MalH.

The *malEFG* operon is known to be subjected to RNase E-mediated processing [54], which led us to hypothesize that MalH is produced through endonucleolytic cleavage of *malG*. To test this, Northern blot analyses were performed with a *malG* 3ʹUTR probe to detect not only MalH, but also any processed *malEFG* RNA fragments containing this region. For this experiment, we used RNA extracted from cells harvested at different optical densities. We detected many shorter *malG* 3ʹUTR-containing RNA fragments including a very short and abundant RNA species that peaks at an optical density of 1.8, which we speculated could be MalH (Fig. 3C). RNase E cleavage was detected 10 nucleotides downstream of the site identified in MalH in the 3ʹUTR of *malG* in the *Salmonella* TIER-seq data [55] by primer extension (Supplementary Figure 1A). If MalH is indeed produced by RNase E cleavage, then the expectation would be that it has a 5ʹ-monophosphate and therefore be susceptible to 5ʹ-monophosphate dependent exonuclease (TEX) degradation. Consistent with this idea, data from published 5ʹ end mapping analyses that used TEX to identify processed RNAs, supported the existence of a short RNA in the same region that has a 5ʹ-monophosphate [56]. In this analysis, the 5ʹ end of MalH was quite heterogeneous; however, a potential RNAse E cleavage site was located 4 nucleotides downstream of the site mapped by primer extension (Supplementary Figure 1A). To confirm that MalH indeed has a 5ʹ monophosphate, we performed Northern blot analyses on RNA samples that were incubated with TEX (Fig. 3D). MalH could not be detected in our TEX treated RNA samples (Fig. 3D, lanes 2 and 4), confirming that it bears a 5ʹ monophosphate. The positive control CpxQ [27] was also degraded in the presence of TEX (Fig. 3D). In contrast, the independently transcribed RybB sRNA was a poor substrate for the exonuclease (Fig. 3D). Note that although the 5S rRNA has a 5ʹ monophosphate it is not degraded by TEX. TEX treatment of total RNA also reduced the levels of the longer intermediate species. These data support a previously described mechanism by which the full-length polycistronic RNA undergoes decay that is initiated at a site in the upstream *malEFG* region. The distal gene *malE* is clipped off by the degradosome and selectively stabilized, allowing its expression at higher levels than other members of the operon [54]. The *malG* 3ʹ end, however, would presumably be less susceptible to further degradation because it is stabilized by Hfq binding.

### MalH directly regulates the expression of major outer membrane

**Figure 4.**
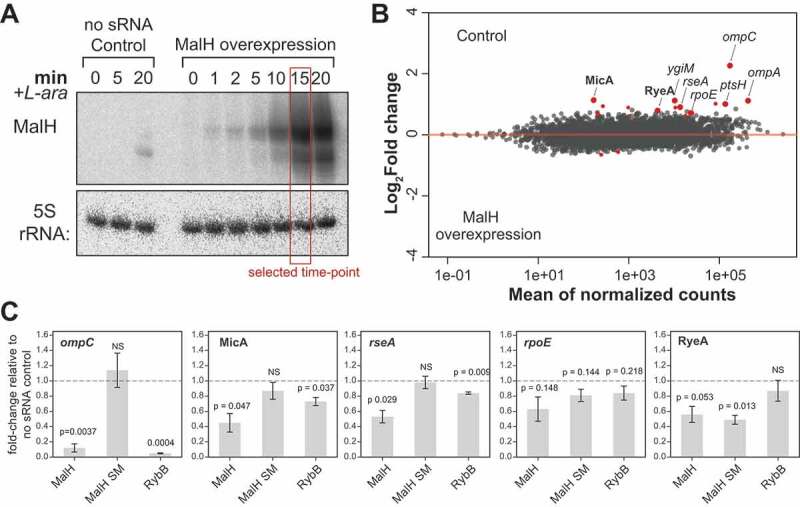
MalH destabilizes *ompC* and *ompA* mRNAs and downregulates key members the σ^E^ regulon

To verify the MalH CLASH data we pulse over-expressed MalH under the control of an arabinose inducible promoter followed by RNA sequencing (Fig. 4A-B). Northern blot analyses of an arabinose time-series experiments revealed that MalH was maximally expressed already after 15 minutes of induction. Hence, to minimize secondary changes in gene expression, cells were harvested at this time-point and the resulting RNA was analysed by RNA sequencing (Fig. 4A, lane labelled with red rectangle). Note that the induction was performed at OD_600_ = 0.4, when endogenous levels of MalH (the band visible by Northern blot in the no sRNA control samples; Fig. 4A) are very low. Differential gene expression analysis identified ~20 transcripts that were significantly reduced upon MalH over-expression (Fig. 4B; Supplementary Table 1), implying these transcripts are regulated by MalH *in vivo*. This set of transcripts included those encoded by the sigma factor RpoE (σ^E^) and the anti-σ^E^ protein RseA. Both are members of the same operon and play an important role in controlling gene expression during stress responses, including the envelope stress response [57–60].

(A) Pulse over-expression of MalH using L-arabinose (see Materials and Methods for details). The empty pBAD plasmid served as a negative control. For the RNA-seq analyses, samples were harvested by rapid filtration 15 min after induction (indicated by the red box). Note that the over-expressed MalH is slightly longer than the endogenous MalH. We believe this is because of RNA polymerase readthrough at the MalH transcriptional terminator, which is then terminated at the transcription terminator present in the plasmid (B) MalH regulates expression of various mRNAs and sRNAs. DESeq2 analyses were performed on RNA-seq data from three biological replicates (Supplementary Table 1). Red points indicate differentially expressed transcripts. Transcripts with a log_2_ fold-change >0 were enriched in the Control data; those with a log_2_ fold-change <0 were enriched in the MalH over-expression data. The annotated, enlarged red data points indicate several differentially expressed transcripts, some of which are discussed in the text. (C) The MalH seed region is important for target regulation. RT-qPCR analysis of several differentially expressed transcripts (gene names shown at the top of the plots), in the presence of sRNAs. MalH SM is MalH with mutations in ‘stem 1’ of the seed region (Fig. 1C and Supplementary Fig. 2C). The plasmid-borne sRNAs were induced for 15 minutes using L-arabinose. The sRNA names are indicated below each bar; the ‘no sRNA’ sample contains the empty plasmid as reference for fold-change calculations; *recA* was used as the internal reference gene; experiments were performed in technical triplicates; the standard error of the mean (SEM) of three biological replicates are reported as error bars. The significance of the differences between the ‘no sRNA’ control and MalH or MalH SM was assessed with a one-sample Student’s t-test. Source data for the RT-qPCR data are provided in the Source Data file.

Intriguingly, MalH over-expression also reduced the levels of sRNAs RyeA and MicA, the latter depending on RpoE (σ^E^) for its expression [61]. In *Salmonella*, MicA downregulates LamB, a high-affinity maltose/maltodextrin transporter [20]. Fragments of three mRNAs (*ompC, ompA* and *ptsH*) that were found in MalH chimeric reads were also differentially expressed, providing strong evidence that these are direct MalH targets.

To substantiate these analyses, we repeated the MalH over-expression experiments and performed RT-qPCR to verify some of the results from the differential expression analyses (Fig. 4C). For this experiment, we also included a MalH mutant in which the seed sequence (stem 1 sequence in Fig. 1C and Supplementary Fig. 2C) was changed into its complementary sequence (referred to as MalH SM mutant). As control, we also included the RybB sRNA, which regulates *rpoE* and *ompC* expression [21,22,24]. These results suggest that MalH, but not the seed mutant, can significantly reduce the levels of *ompC*, MicA and *rseA* (Fig. 4C). Although the qPCR data were generally in agreement with the RNA-seq data, we could not reproducibly detect significant changes in *rpoE* mRNA levels upon MalH over-expression. To our surprise, we did not observe significant changes in *rpoE* mRNA levels upon RybB over-expression, while levels of another target (*ompC*) were dramatically reduced. Notably, over-expression of the MalH seed mutant also resulted in a significant reduction in RyeA levels, implying that the observed changes are not a result of direct interactions between MalH and RyeA or that the regulation involves regions of MalH located outside of the main seed region.

**Figure 5.**
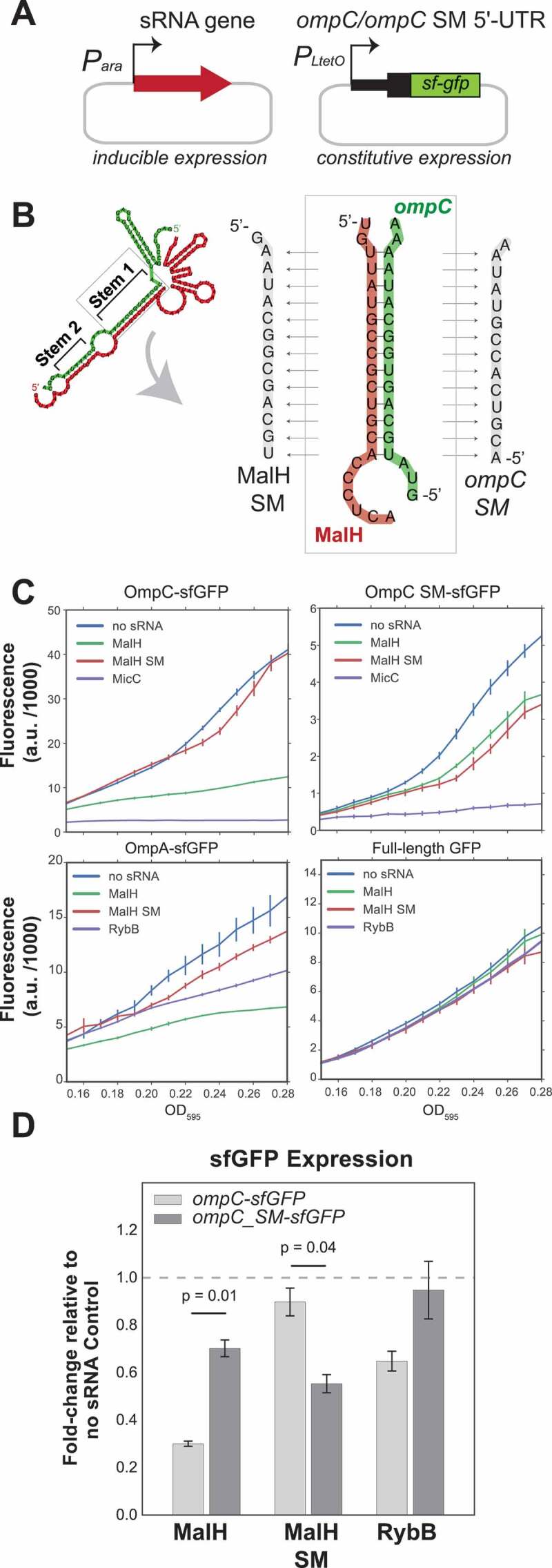
Validation of MalH-*ompC* interaction using GFP reporters

To demonstrate the direct target regulation *in vivo*, we employed a well-established reporter system where an sRNA is co-expressed with a construct containing the mRNA target region fused to the coding sequence of superfolder green fluorescent protein (sfGFP) [19,39] (Fig. 5A). Fusions for OmpC, OmpA and σ^E^ were constructed, but only the OmpC and OmpA-sfGFP reporters produced stable fusions that could be analysed. We also included a MalH sRNA seed sequence mutant (MalH SM) and an *ompC* mutant containing compensatory mutations (OmpC SM; Fig. 5B). Importantly, the seed mutations in MalH did not affect the stability of the sRNA expressed from pBAD (Supplementary Fig. 3A and B). On the contrary, our OmpA-GFP reporter construct containing compensatory mutations in the target region did not generate a stable fusion. Therefore, we were unable to use this reporter system to fully verify the MalH-*ompA* interaction. As positive controls, we used sRNAs MicC and RybB sRNAs as they both regulate *E. coli ompC* expression [21,62]. Fluorescence measurements confirmed that levels of OmpC-sfGFP and OmpA-sfGFP fusions were significantly lower in cells expressing MalH (Fig. 5C). Importantly, MalH over-expression did not change the expression of the GFP reporter itself (Fig. 5C). Mutations in the MalH seed region largely restored OmpA- and OmpC-sfGFP reporter levels to the levels of the no sRNA negative control. The MalH SM mutant partially restored the expression of the OmpC SM-sfGFP mutant, suggesting that the base-pairing interaction between these two mutants is less stable compared to the wild-type (Fig. 5C). As expected, MicC was able to suppress OmpC SM-sfGFP levels as it base-pairs with the Stem 2 sequence in *ompC* (Supplementary Fig. 2C). The wild-type MalH was also able to partially suppress the expression of the OmpC SM mutant. We suggest that the predicted base-pairing interactions between MalH and *ompC* mRNA in the second stem (Supplementary Fig. 2C; ‘stem 2’) might be sufficient to at least partially suppress *ompC* expression. Regardless, the data strongly imply that MalH directly regulates *ompC* expression.

(A) Plasmid system used for the reporter assay: in *E. coli* TOP10 cells, low-copy plasmids constitutively over-express target 5ʹUTRs fused to sfGFP and medium-copy plasmids over-express the full-length sRNAs upon induction with L-arabinose. (B) Design of the wild-type and mutant *ompC* constructs. The panel indicates the base-pairing region within the MalH-*ompC* duplex that was mutated. We created an MalH seed mutant (SM) and an *ompC* mutant in which base pairing with MalH SM was restored. (C) MalH downregulates expression of OmpC and OmpA sfGFP fusions. *In vivo* fluorescence measurements of OmpC, OmpC SM and OmpA sfGFP fusion proteins were measured using a Tecan plate reader system at OD_595_. As a negative control, we included sfGFP alone in the presence or absence of sRNAs. The ‘no sRNA’ expressing strains contain the empty pBAD plasmid. The y-axis indicates fluorescence units (F.U.) reported by the plate reader. Experiments were performed in technical and biological triplicates; the fluorescence means and SEM of three biological replicates are reported. Source data are provided as a Source Data file. (**D**) MalH directly downregulates *ompC* mRNA through base-pairing interactions: RT-qPCR analyses of the *ompC-sfGFP* and *ompC SM-sfGFP* fusions expression in the presence of MalH. Cells were grown in LB to OD_600_ of ~0.4, induced with arabinose and harvested at OD_600_ 0.6–0.7. The bars indicate the mean fold-change in expression relative to the no sRNA Control (horizontal dashed line). Error bars indicate the standard error of the mean from two biological replicates. The significance of the differences between the WT and the MalH seed mutant (MalH SM) was assessed with a two-tailed Student’s t-test.

To determine whether the changes in fluorescence were associated with changes in reporter mRNA levels, we measured the expression of the GFP reporters by RT-qPCR. The results were essentially identical to the GFP fluorescence measurements (Fig. 5D). Over-expression of the wild-type MalH, but not of the seed mutant, reduced *ompC*-sfGFP mRNA levels. The *ompC* seed mutation (SM) did not fully disrupt the regulation by wild-type MalH. However, the MalH mutant containing compensatory mutations (SM mutant) was able to suppress the *ompC* SM mRNA levels, consistent with the idea that base-pairing was partially restored.

**Figure 6.**
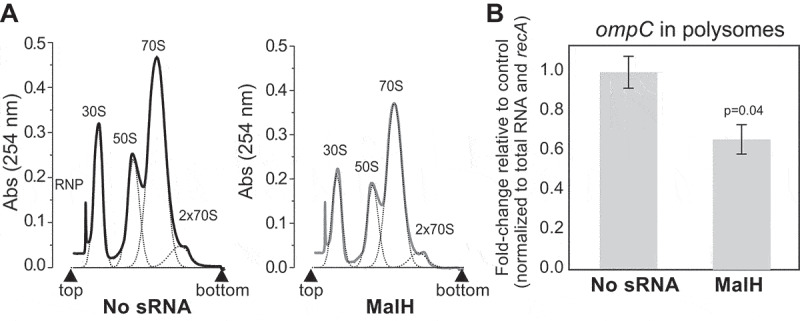
MalH regulates *ompC* mRNA translation in *E. coli.*

Next, we performed polysome profiling experiments to assess the level of *ompC* recruitment on polysomes upon over-expression of MalH. Over-expression of MalH did not noticeably affect 70S and polysome levels (Fig. 6A). We observed a significant (~37%) reduction of *ompC* mRNA in the polysomal fractions, relative to the upper fractions (Fig. 6B). We hypothesize that MalH regulates *ompC* expression at the post-transcriptional and translational level.

(A) Cultures at OD_600_ 0.4 over-expressing MalH or no sRNA for 15 minutes were harvested by rapid filtration and subjected to polysome profiling. Profiles of the polysomal (2x70S) and subpolysomal fractions obtained for the empty plasmid control and MalH over-expression samples. (B) The RT-qPCR analysis of the polysomal fractions: A ‘total’ fraction was obtained by mixing equal volumes of the polysomal and subpolysomal fractions and is representative of the lysate content. Expression of *ompC* in the polysomal fractions was quantified relative to the amount in ‘total’ RNA, normalized to *recA*, and calculated as fold-change relative to the control sample (y-axis). The RT-qPCR experiments were performed in biological and technical triplicates; the standard error of the mean (SEM) of three biological replicates fold changes are reported as error bars. Significance of the difference in *ompC* mRNA level in polysomes was assessed with a two-tailed Student’s t-test. Source data for the RT-qPCR data are provided in the Source Data file.

### MalH tunes maltoporin expression during maltose fermentation

**Figure 7.**
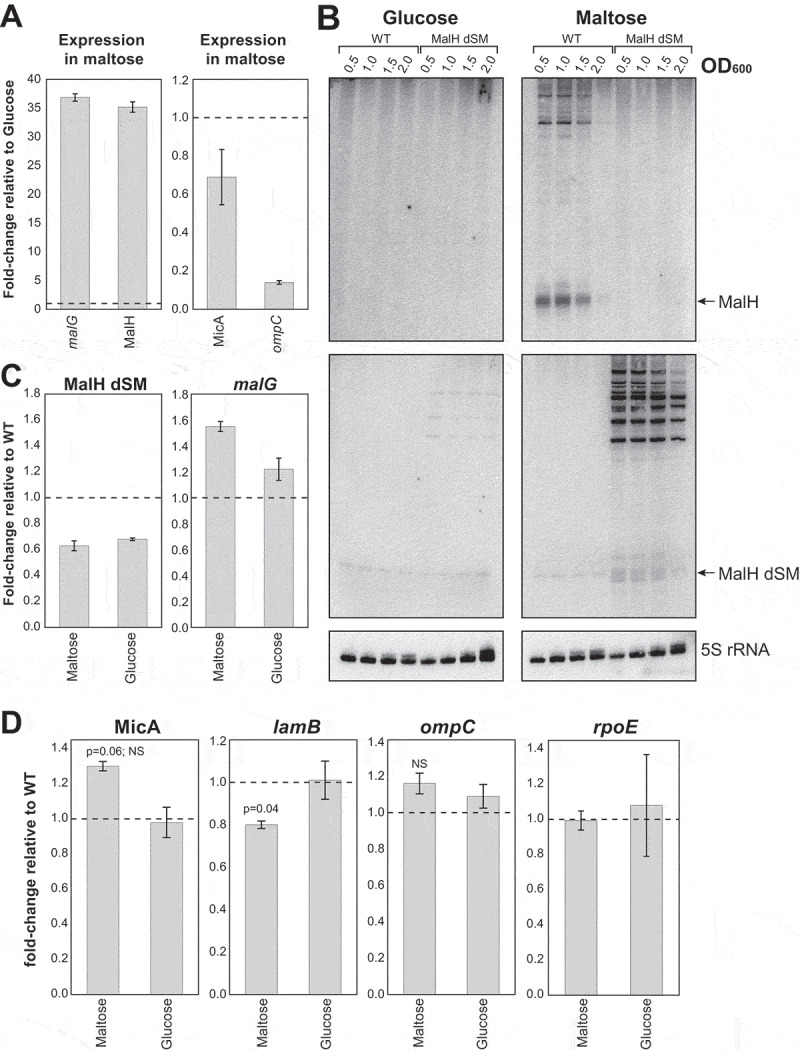
MalH modulates expression of key factors involved in maltose intake

**Figure 8.**
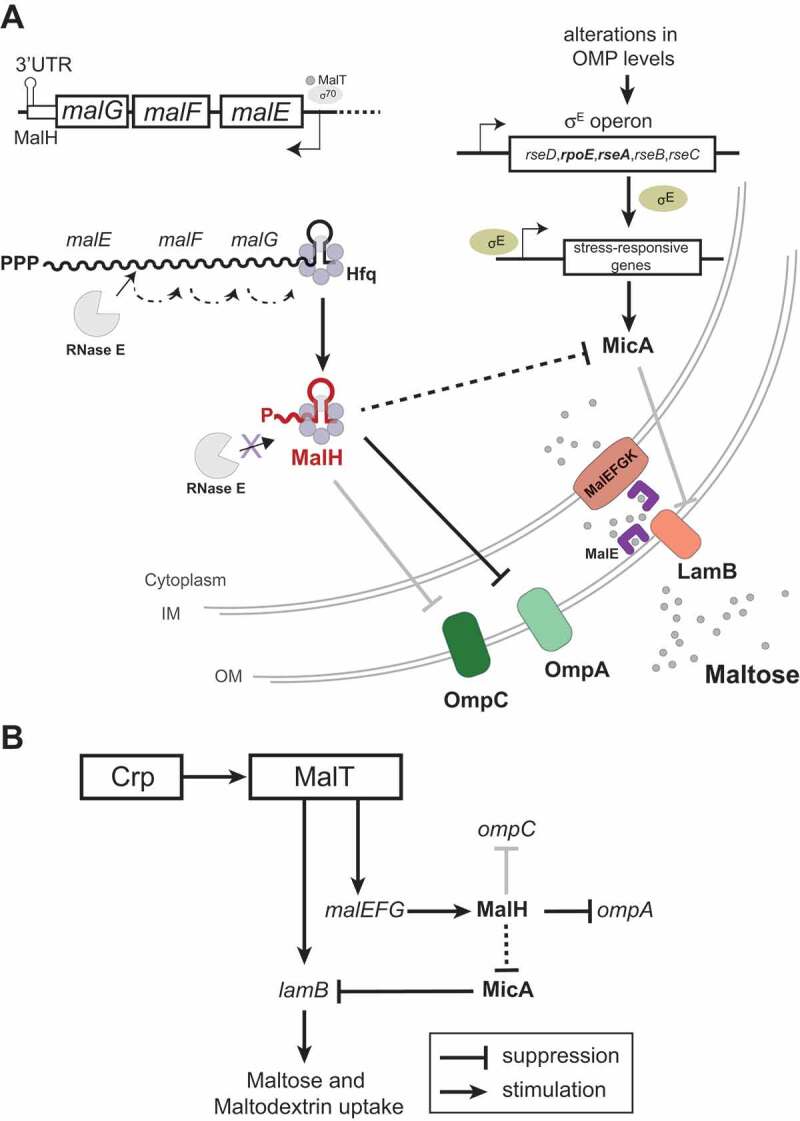
An sRNA-target interaction networks that contributes to adaptation to nutrient availability

Considering that MalH is produced from the *malEFG* operon, we reasoned that the novel sRNA may be involved in adaptation to growth in maltose containing medium. Because our previous analyses demonstrated that mutating the stem 1 sequence in MalH did not completely disrupt the regulation of *ompC* (Fig. 5), we generated a strain in which the predicted base-pairing interactions of MalH with its targets (both stem 1 and 2; Supplementary Fig. 2C and Fig. 5B; MalH dSM (double seed mutant)) was disrupted. Subsequently, overnight cultures grown in glucose were split and (re)inoculated in fresh medium containing either glucose or maltose as the sole carbon sources, and expression of several *mal* regulon genes and MalH targets were quantified. We show that MalH and its parental transcript *malG* are almost undetectable during growth in glucose, and relatively highly expressed during growth in maltose (~35-fold increase, Fig. 7A and 7B). This is consistent with catabolite repression of the *mal* regulon by glucose, and its induction by maltose [38]. Intriguingly, we observed that *ompC* mRNA levels are overall significantly lower during growth in maltose, compared to glucose (Fig. 7A). This suggests that porin expression is also regulated by nutrient source in *E. coli*. Similarly, levels of MicA, a repressor of LamB synthesis [20,21], were reduced by ~30% in maltose compared to glucose (Fig. 7A). Notably, Northern blot (Fig. 7B) and qPCR (Fig. 7C) analyses revealed that when cells are grown in maltose, the MalH dSM mutant less abundant than the wild-type but longer processed fragments that contained upstream *malG* regions could still be detected (Fig. 7B). Although the main RNase E cleavage site was not mutated in the MalH dSM mutant, it appears that the mutations partially blocked MalH processing, which could impact the half-life of the *malG* mRNA as well. Unfortunately, because the probe designed to detect both MalH WT and MalH dSM detected only shorter MalH degradation intermediates but not the longer *malEFG* degradation intermediates (Supplementary Fig. 4; Supplementary Table 4; MalG_3ʹUTR), we were unable to accurately quantify to what extent processing of the MalH was impaired. As a compromise, we measured *malG* and *malH* levels by RT-qPCR (Fig. 7C). From these results, we estimated that in the MalH dSM strain *malG* levels increased roughly 1.6-fold and MalH levels decreased ~40% in maltose. To measure the impact of these MalH seed mutations, we first compared the expression levels of *ompC*, MicA, and *lamB* between the wild type and MalH dSM strains. In maltose, MicA levels increased by roughly 30% in the MalH dSM seed-mutant strain (Fig. 7D). Concomitantly, *lamB* levels were reduced by ~20% in this medium. *OmpC* expression did not significantly change in the mutant in either maltose or glucose (Fig. 7D). Because MalH over-expression slightly (but not significantly) reduced σ^E^ mRNA levels (*rpoE*; Fig. 4B-C) and σ^E^ controls the synthesis of MicA, we also analysed this mRNA by RT-qPCR in these strains. In the mutant strain, *rpoE* levels also did not significantly change in maltose, corroborating that MalH does not directly control its transcript levels (Fig. 7D). Based on these results, we hypothesize that when cells need to use maltose as a main carbon source, subsequent MalH expression enhances the uptake of maltose by (directly or indirectly) reducing MicA levels, independently of *rpoE*. This in turn could enhance the production of the LamB maltoporin (Figs. 7D and 8A). However, because the seed mutations also affected the synthesis of MalH, we cannot completely rule out the possibility that the observed changes in *lamB* and MicA levels were, at least in part, the result of defective RNase E cleavage of *malG*.

(A) Endogenous MalH and *malG* expression is induced during growth on maltose, and *ompC* levels are significantly lower in maltose compared to glucose. Total RNA extracted from exponentially growing cells (OD_600_ 0.5) and MalH, *malG* (Left), and MalH direct (*ompC*) and indirect (MicA) targets was quantified by RT-qPCR. The data were normalized to 5S rRNA levels. The bars indicate the mean fold-change in expression relative to expression in cells growing in glucose (indicated on the plot with a horizontal-dashed line). Error bars indicate the standard error of the mean from two biological replicates. (B) MalH seed sequences are important for RNase E-recruitment and MalH biogenesis. Northern blot that compares MalH and longer *malG*-3ʹUTR containing fragments expression in wild-type *E. coli* and the MalH seed-mutant strain (MalH dSM). (C) The MalH dSM mutant is ~40% less abundant than the wild-type. Cell growth and RT-qPCR analysis of MalH, MalH dSM and *malG* expression were performed as in (A). The bars indicate the mean fold-change in expression relative to the wild-type. Error bars indicate the standard error of the mean from two biological replicates. (D) Increased MicA and decreased *lamB* levels in the MalH dSM mutant but *ompC* and *rpoE* do not significantly change. The bars indicate the mean fold-change in expression relative to the wild-type. Error bars indicate the standard error of the mean from two biological replicates. P-values were calculated using a one-sample t-test. Source data for the RT-qPCR data are provided in the Source Data file.

(A) Model for MalH biogenesis and its role in post-transcriptional regulation. During maltose (grey circles) utilization, MalT transcribes the *malEFG* operon that encodes components for maltose uptake. A fraction of the *malEFG* transcripts is degraded by RNase E, generating the 3ʹUTR-derived MalH sRNA. MalH base-pairs with *ompC* mRNA, leading to its decay. MalH downregulates (either directly or indirectly) MicA that represses LamB synthesis. Additionally, MalH may downregulate expression of outer membrane points *ompC* and *ompA*. Therefore, as part of a feed-forward loop (FFL), MalH promotes accumulation of maltose-specific porins, which facilitates maltose and maltodextrin uptake. (B) MalT transcribes *lamB* and, via *malEFG* transcription, promotes MalH accumulation. MalH indirectly promotes LamB synthesis by repressing MicA. Thus, MalT and MalH join both promote *lamB* expression, forming a mixed coherent FFL. The grey lines indicate interactions that were detected by Hfq CLASH and RIL-seq [33,34] and experimentally verified interactions either by other groups (MicA and *lamB* [20]) or in this work (MalH, *ompC*). The bold black line connecting MalH and OmpA indicates that the interaction was detected in Hfq CLASH and RIL-seq data [33,34], verified by over-expression but not fully verified by our GFP reporter experiments. Dashed lines indicate likely indirect interactions.

## Discussion

Our previous Hfq CLASH study [33] identified over 100 distinct interactions between 3ʹUTRs and other mRNA regions, which implicate direct mRNA-mRNA communication. We proposed that many of these represent novel 3ʹUTR-derived sRNAs that target 5ʹUTRs of mRNAs. MalH is a new sRNA that was uncovered by Hfq CLASH and RIL-seq and consists entirely of the 3ʹUTR of the *malG* transcripts. This sRNA was of particular interest as it was mainly detected during the transition from late exponential to early stationary phase. Here we propose that MalH plays a role in improving maltose uptake by enhancing expression of the high-affinity maltose transporter LamB (Fig. 8A).

For our studies, we used Lysogeny broth (LB). LB is a well-defined medium that is rich in peptides and has a low content of utilizable sugars [9]. Although catabolisable amino acids are the main carbon source that support growth in LB [9], LB also contains sugar mixtures in short supply, which made it an ideal medium for our studies on nutrient limitation. These sugars are consumed one after another or co-utilized in *E. coli* cultures depending on their hierarchy and concentration [5,10,63]. Glucose, the preferred carbon source, is consumed first, followed by other fermentable sugars such as maltodextrins, nucleotides, and free sugars. During growth in LB, glucose is utilized during the exponential phase when the *mal* regulon genes are not expressed due to catabolite repression [38]. Glucose depletion is followed by simultaneous activation of systems for the utilization of non-glucose carbon sources. Accordingly, proteomics studies have shown that the entry into stationary phase in LB is characterized by increased expression of proteins required for scavenging nutrients and the uptake of alternative carbon sources: proteins of the phosphotransferase (PTS) system, maltose, amino acid and peptide transporters [64]. Studies in LB have shown that maltodextrins are the first substrates to support growth after glucose limitation [5]. Maltose/maltodextrins need to be transported into cells before their use in catabolic and anabolic processes. Efficient uptake of these carbon sources involves the maltose-specific transport system, which includes inner membrane, periplasmic and outer membrane (OMP) components encoded by *malEFG* and *lamBmalK* operons, respectively (Fig. 8A). The MalT transcription factor bound by maltotriose, induces transcription of genes encoding maltose transporters and *mal* enzymes. Although *malEFG* genes are transcribed as a single unit, RNase E processing of the polycistronic transcript can generate a differential expression of the monocistronic mRNAs [65]. Here we show that the MalH sRNA is a product of *malEFG* processing, which we hypothesize is stabilized by Hfq binding.

The induction of the Mal system and MalH during the transition phase in LB is a cumulative effect of glucose limitation, increased levels of cAMP and CRP, exogenous induction and synthesis of maltotriose endoinducer. By monitoring the expression of the targets of MalH during growth on maltose as the sole carbon source, we were able to specifically ascribe a hypothetical role to MalH in the optimization of exogenous maltose uptake. MalH is unique in a sense that it is not only an mRNA-derived sRNA targeting multiple pathways, but it is also because it is part of a ‘mixed coherent feed forward loop’ (FFL [66]) that we predict promotes maltose uptake via LamB. A major advantage of a coherent FFL is that it can accelerate or delay responses to stimuli, such as changes in carbon source availability. In this FFL, the key activator is the MalT transcription factor, which induces the expression of both mal*EFG* and *lamB* when cells start to consume maltose (Fig. 8B). We propose that *malEFG* indirectly promotes LamB accumulation via the MalH sRNA by (directly or indirectly) reducing the expression of MicA. This downregulation is important as MicA interferes with translation of the *lamB* mRNA through base-pairing interactions [20]. Consistent with this model, in the MalH seed-mutant MicA was expressed at ~30% higher levels. Although a 15 min over-expression of MalH was not sufficient to significantly impact *lamB* levels (Supplementary Table 1), mutating the MalH seed sequences in the genome did reduce *lamB* levels by ~20% (Fig. 7D). While these changes appear modest, it is important to take into consideration the very high abundance of the *lamB* mRNAs. MicA expression is controlled by the sigma factor *rpoE* (σ^E^) and we see a modest reduction of *rpoE* and *rseA* mRNA levels from the σ^E^ operon upon over-expression of MalH (Fig. 4C). This raises the possibility that MalH indirectly suppresses MicA by downregulating *rpoE*. However, the strain carrying mutations in the MalH seed sequence did not show significant changes in *rpoE* mRNA levels. Direct downregulation of MicA by MalH, however, is less likely as no evidence for base-pairing between these sRNAs was found in either our Hfq CLASH [33] data or by computational prediction. Furthermore, besides *ompA* and *ompC*, other mRNAs directly regulated by MicA (*tsx, ygiM*) were downregulated upon MalH over-expression (Fig. 4B, Supplementary Table 1) which would be expected to have gone up if MalH acted directly on MicA. Some of these genes are also regulated by other sRNAs, such as MicC (*ompC*), RybB (*tsx, ompC, ompA*), or RseX (*ompA, ompC*) [62,67,68] (Supplementary Fig. 2B-C), while the RNA binding protein CsrA, a carbon storage regulator, represses *rpoE* expression [69]. Therefore, it is possible that MalH acts in concert with these other post-transcriptional regulators to control the expression of these differentially expressed genes.

Our data imply that MalH base-pairs with the *ompC* mRNA and, when over-expressed, MalH can effectively reduce its steady-state levels. However, in medium with maltose as sole carbon source, endogenous MalH expression was not sufficient to significantly affect *ompC* mRNA levels. However, given the very high abundance and stability of *ompC* (minutes-long half-life [70]), we predict that even a mild reduction in *ompC* levels can profoundly relieve the pressure on the translation machinery and OMP assembly pathways [30]. The potential dual regulation by MalH (repression of OmpC and upregulation of LamB) could help balance levels of OMPs in response to changes in the available nutrients (Fig. 8A). Although this model is intriguing, the biological significance of MalH remains unclear. Interestingly, maltose has been shown to be a key nutrient for efficient colonization of mouse intestines by pathogenic and commensal strains of *E. coli* [71,72]. It would therefore be interesting to see if the MalH seed mutant has a competitive disadvantage in intestinal colonization.

MalH is reminiscent of the *Vibrio cholerae* MicX sRNA that also maps to the 3ʹ region of *malG* and regulates levels of outer membrane proteins [73]. However, the MicX targets (uncharacterized OMP and a peptide ABC transporter) are different from the MalH ones. Furthermore, unlike MalH, MicX is produced from an independent promoter. Conditions that change the expression of the upstream Mal operon in *V. cholerae* do not affect accumulation of MicX [73], whereas the levels of MalH strongly correlate with *malEFG* mRNA levels. Nevertheless, both studies demonstrate that an sRNA resides in the 3ʹUTR of the *malG* transcript and pinpoints a mechanism where the expression of multiple porins is connected by sRNAs encoded within transporter mRNAs.

Analysis of the Hfq CLASH and RIL-seq data revealed that MalH could also potentially control sRNA levels or activity. For example, we frequently recovered chimaeras that contained MalH fragments fused to fragments of a *cis*-encoded sRNA, OhsC. OhsC is an sRNA that is part of a toxin-antitoxin system that suppresses the production a short hydrophobic and toxic protein (ShoB) of which the expression needs to be tightly controlled [74,75]. It is tempting to speculate that MalH indirectly controls ShoB expression by sponging OhsC. *Vice versa*, OhsC could also control MalH activity/stability.

We also found interactions between MalH and transcripts that are part of the *pts* operon (*ptsI* and *ptsH* [76]) in both RIL-seq and Hfq CLASH data. This operon codes for three components of the phosphoenolpyruvate-dependent phosphotransferase system (PTS) that regulates the uptake, phosphorylation and metabolism of a number of energetically preferred sugars (PTS sugars) [77], such as glucose. Uptake of non-PTS sugars, such as maltose and maltodextrins, is regulated by the EIIA^glc^ enzyme. In the presence of an excess of glucose, this enzyme accumulates in an unphosphorylated state, which blocks maltose/maltodextrin uptake by binding to the MalK homodimer (Fig. 8A) [78]. PtsI and PtsH are non-sugar specific components of the PTS system that can phosphorylate EIIA^glc^. Thus, we hypothesize that MalH post-transcriptionally enhances the production of PtsI and PtsH (presumably by stimulating translation) to maintain sufficiently high levels of phosphorylated EIIA^glc^.

In conclusion, our work provides an intriguing example of how an mRNA-derived sRNA can enhance the output of its regulon by not only dampening pathways that inhibit the accumulation of the encoded maltose transporters but possibly also by regulating the enzymes that control the activity of the maltose transporters.

## Supplementary Material

Supplemental Material

## References

[1] El Mouali Y, Gaviria-Cantin T, Sánchez-Romero MA, et al. CRP-cAMP mediates silencing of Salmonella virulence at the post-transcriptional level. PLoS Genet. 2018;14(6):1–26. 10.1371/journal.pgen.1007401 PMC5991649 29879120

[2] Peyraud R, Cottret L, Marmiesse L, et al. Control of primary metabolism by a virulence regulatory network promotes robustness in a plant pathogen. Nat Commun. 2018;9. 10.1038/s41467-017-02660-4 29379078 PMC5788922

[3] Luckett JCA, Darch O, Watters C, et al. A novel virulence strategy for pseudomonas aeruginosa mediated by an autotransporter with arginine-specific aminopeptidase activity. PLoS Pathog. 2012;8(8):e1002854. . 10.1371/journal.ppat.1002854 PMC3426542 22927813

[4] Anderson MT, Mitchell LA, Zhao L, et al. Capsule production and glucose metabolism dictate fitness during serratia marcescens bacteremia. MBio. 2017;8(3). DOI:10.1128/mBio.00740-17 PMC5442460 28536292

[5] Baev MV, Baev D, Radek AJ, et al. Growth of Escherichia coli MG1655 on LB medium: monitoring utilization of sugars, alcohols, and organic acids with transcriptional microarrays. Appl Microbiol Biotechnol. 2006;71(3):310–316. 16628448 10.1007/s00253-006-0317-6

[6] Baev MV, Baev D, Radek AJ, et al. Growth of Escherichia coli MG1655 on LB medium: determining metabolic strategy with transcriptional microarrays. Appl Microbiol Biotechnol. 2006;71(3):323–328. 16645822 10.1007/s00253-006-0392-8

[7] Baev MV, Baev D, Jansco Radek A, et al. Growth of Escherichia coli MG1655 on LB medium: monitoring utilization of amino acids, peptides, and nucleotides with transcriptional microarrays. Appl Microbiol Biotechnol. 2006;71(3):317–322. 16575570 10.1007/s00253-005-0310-5

[8] Ferenci T. Regulation by nutrient limitation. Curr Opin Microbiol. 1999;2(2):208–213. 10322163 10.1016/S1369-5274(99)80036-8

[9] Sezonov G, Joseleau-Petit D, D’Ari R. Escherichia coli physiology in Luria-Bertani broth. J Bacteriol. 2007;189(23):8746–8749. 17905994 10.1128/JB.01368-07 PMC2168924

[10] Ferenci T. Adaptation to life at micromolar nutrient levels: the regulation of Escherichia coli glucose transport by endoinduction and cAMP. FEMS Microbiol Rev. 1996;18(4):301–317. 8703508 10.1111/j.1574-6976.1996.tb00246.x

[11] Kenyon WJ, Thomas SM, Johnson E, et al. Shifts from glucose to certain secondary carbon-sources result in activation of the extracytoplasmic function sigma factor $σ$E in Salmonella enterica serovar Typhimurium. Microbiology. 2005;151(7):2373–2383. 16000727 10.1099/mic.0.27649-0 PMC1489810

[12] Hör J, Matera G, Vogel J, et al. Trans-acting small RNAs and their effects on gene expression in escherichia coli and salmonella enterica. EcoSal Plus. 2020;9(1). DOI:10.1128/ecosalplus.ESP-0030-2019 PMC7112153 32213244

[13] De Mets F, Van Melderen L, Gottesman S. Regulation of acetate metabolism and coordination with the TCA cycle via a processed small RNA. Proc Natl Acad Sci. 2018. 10.1073/pnas.1815288116. PMC6338826 30591570

[14] Miyakoshi M, Matera G, Maki K, et al. Functional expansion of a TCA cycle operon mRNA by a 3′ end-derived small RNA. Nucleic Acids Res. 2018;47(4):2075–2088. 10.1093/nar/gky1243 PMC6393394 30541135

[15] Rice JB, Vanderpool CK. The small RNA SgrS controls sugar-phosphate accumulation by regulating multiple PTS genes. Nucleic Acids Res. 2011;39(9):3806–3819. 21245045 10.1093/nar/gkq1219 PMC3089445

[16] Beisel CL, Storz G. The base-pairing RNA Spot 42 participates in a multioutput feedforward loop to help enact catabolite repression in Escherichia coli. Mol Cell. 2011;41(3):286–297. 21292161 10.1016/j.molcel.2010.12.027 PMC3072601

[17] Urbanowski ML, Stauffer LT, Stauffer GV. The gcvB gene encodes a small untranslated RNA involved in expression of the dipeptide and oligopeptide transport systems in Escherichia coli. Mol Microbiol. 2000;37(4):856–868. 10972807 10.1046/j.1365-2958.2000.02051.x

[18] Pulvermacher SC, Stauffer LT, Stauffer GV. The small-RNA GcvB regulates sstT mRNA expression in Escherichia coli. J Bacteriol. 2009;91(1):238–248. 10.1128/JB.00915-08 PMC2612445 18952787

[19] Urban JH, Vogel J. Translational control and target recognition by Escherichia coli small RNAs in vivo. Nucleic Acids Res. 2007;35(3):1018–1037. 17264113 10.1093/nar/gkl1040 PMC1807950

[20] Bossi L, Figueroa-Bossi N. A small RNA downregulates LamB maltoporin in Salmonella. Mol Microbiol. 2007;65(3):799–810. 17608792 10.1111/j.1365-2958.2007.05829.x

[21] Gogol EB, Rhodius VA, Papenfort K, et al. Small RNAs endow a transcriptional activator with essential repressor functions for single-tier control of a global stress regulon. Proc Natl Acad Sci U S A. 2011;108(31):12875–12880. 21768388 10.1073/pnas.1109379108 PMC3150882

[22] Papenfort K, Pfeiffer V, Mika F, et al. σE-dependent small RNAs of Salmonella respond to membrane stress by accelerating global omp mRNA decay. Mol Microbiol. 2006;62(6):1674–1688. 17427289 10.1111/j.1365-2958.2006.05524.x PMC1804206

[23] Johansen J, Rasmussen AA, Overgaard M, et al. Conserved small non-coding RNAs that belong to the sigmaE regulon: role in down-regulation of outer membrane proteins. J Mol Biol. 2006;364(1):1–8. 17007876 10.1016/j.jmb.2006.09.004

[24] Thompson KM, Rhodius VA, Gottesman S. σE regulates and is regulated by a small RNA in Escherichia coli. J Bacteriol 2007; 189(11):4243–4256. 17416652 10.1128/JB.00020-07 PMC1913397

[25] Tree JJ, Granneman S, McAteer SP, et al., Identification of bacteriophage-encoded anti-sRNAs in pathogenic Escherichia coli. Mol Cell. Internet] 2014; 55(2):199–213. 24910100 10.1016/j.molcel.2014.05.006 PMC4104026

[26] Chao Y, Papenfort K, Reinhardt R, et al. An atlas of Hfq-bound transcripts reveals 3′ UTRs as a genomic reservoir of regulatory small RNAs. EMBO J. 2012;31(20):4005–4019. 22922465 10.1038/emboj.2012.229 PMC3474919

[27] Chao Y, Vogel J. A 3′ UTR-derived small RNA provides the regulatory noncoding arm of the inner membrane stress response. Mol Cell. 2016;61(3):352–363. 26805574 10.1016/j.molcel.2015.12.023

[28] Smirnov A, Wang C, Drewry LL, et al. Molecular mechanism of mRNA repression in trans by a ProQ-dependent small RNA. EMBO J. 2017;36(8):1029–1045. 28336682 10.15252/embj.201696127 PMC5391140

[29] Wang C, Chao Y, Matera G, et al. The conserved 3 UTR-derived small RNA NarS mediates mRNA cross-regulation during nitrate. Nucleic Acids Res. 2019;48(4):2126–2143. 10.1093/nar/gkz1168 PMC7038943 31863581

[30] Guo MS, Updegrove TB, Gogol EB, et al. MicL, a new σE-dependent sRNA, combats envelope stress by repressing synthesis of Lpp, the major outer membrane lipoprotein. Genes Dev. 2014;28(14):1620–1634. 25030700 10.1101/gad.243485.114 PMC4102768

[31] Holmqvist E, Li L, Bischler T, et al. Global maps of ProQ Binding In vivo reveal target recognition via RNA structure and stability control at mRNA 3′ Ends. Mol Cell. 2018;70(5):971–982.e6. 10.1016/j.molcel.2018.04.017 29804828

[32] Melamed S, Adams PP, Zhang A, et al. RNA-RNA Interactomes of ProQ and Hfq reveal overlapping and competing roles. Mol Cell. 2020;77(2):411–425.e7. [Internet]. 31761494 10.1016/j.molcel.2019.10.022 PMC6980735

[33] Iosub IA, van Nues RW, McKellar SW, et al. Hfq CLASH uncovers sRNA-target interaction networks linked to nutrient availability adaptation. Elife. 2020;9:1–33. 10.7554/eLife.54655 PMC7213987 32356726

[34] Melamed S, Peer A, Faigenbaum-Romm R, et al. Global mapping of small RNA-target interactions in resource global mapping of small RNA-target interactions in bacteria. Mol Cell. 2016;63(5):884–897. 27588604 10.1016/j.molcel.2016.07.026 PMC5145812

[35] Grabowicz M, Koren D, Silhavy TJ. The CpxQ sRNA negatively regulates Skp To Prevent Mistargeting of ␤ -barrel outer membrane proteins into the cytoplasmic membrane. MBio. 2016;7(2):1–8. 10.1128/mBio.00312-16 PMC4817254 27048800

[36] Acuña LG, Barros MJ, Peñaloza D, et al. A feed-forward loop between SroC and MgrR small RNAs modulates the expression of eptB and the susceptibility to polymyxin B in Salmonella typhimurium. Microbiology. 2016;162(11):1996–2004. 27571709 10.1099/mic.0.000365

[37] Miyakoshi M, Chao Y, Vogel J. Cross talk between ABC transporter mRNAs via a target mRNA-derived sponge of the GcvB small RNA. EMBO J. 2015;34(11):e201490546–1492. 10.15252/embj.201490546 PMC4474525 25630703

[38] Boos W, Shuman H. Maltose/maltodextrin system of Escherichia coli: transport, metabolism, and regulation. Microbiol Mol Biol Rev. 1998;62:204–229. 9529892 10.1128/mmbr.62.1.204-229.1998 PMC98911

[39] Corcoran CP, Podkaminski D, Papenfort K, et al. Superfolder GFP reporters validate diverse new mRNA targets of the classic porin regulator, MicF RNA. Mol Microbiol. 2012;84(3):428–445. 22458297 10.1111/j.1365-2958.2012.08031.x

[40] Love MI, Huber W, Anders S. Moderated estimation of fold change and dispersion for RNA-seq data with DESeq2. Genome Biol. 2014;15:550. 25516281 10.1186/s13059-014-0550-8 PMC4302049

[41] Sambrook J, Fritsch EF, Maniatis T. Molecular cloning: A Laboratory Manual, 2nd ed., Vols. 1, 2 and 3. Cold Spring Harbor (NY): Cold Spring Harbor Laboratory Press; 1989.

[42] Tollervey D, Mattaj IW. Fungal small nuclear ribonucleoproteins share properties with plant and vertebrate U-snRNPs. Embo J. 1987;6(2):469–476. 2953599 10.1002/j.1460-2075.1987.tb04777.x PMC553418

[43] Bernabò P, Tebaldi T, Groen EJN, et al. In vivo translatome profiling in spinal muscular atrophy reveals a role for SMN protein in ribosome biology. Cell Rep. 2017;21(4):953–965. 10.1016/j.celrep.2017.10.010 PMC5668566 29069603

[44] Lunelli L, Bernabò P, Bolner A, et al. Peering at brain polysomes with atomic force microscopy. J Vis Exp. 2016;1–8. 10.3791/53851. PMC4829028 27023752

[45] Tebaldi T, Re A, Viero G, et al. Widespread uncoupling between transcriptome and translatome variations after a stimulus in mammalian cells. BMC Genomics. 2012;13(1):220. 10.1186/1471-2164-13-220 PMC3441405 22672192

[46] Datsenko KA, Wanner BL. One-step inactivation of chromosomal genes in Escherichia coli K-12 using PCR products. Proc Natl Acad Sci U S A. 2000;97(12):6640–6645. 10829079 10.1073/pnas.120163297 PMC18686

[47] St-Pierre F, Cui L, Priest DG, et al. One-step cloning and chromosomal integration of DNA. ACS Synth Biol. 2013;2(9):537–541. 10.1021/sb400021j 24050148

[48] Webb S, Hector RD, Kudla G, et al., PAR-CLIP data indicate that Nrd1-Nab3-dependent transcription termination regulates expression of hundreds of protein coding genes in yeast. Genome Biol. 2014; 15:R8. 24393166 10.1186/gb-2014-15-1-r8 PMC4053934

[49] Tjaden B. De novo assembly of bacterial transcriptomes from RNA-seq data. Genome Biol. 2015;16(1). doi:10.1186/s13059-014-0572-2 PMC4316799 25583448

[50] Waterhouse AM, Procter JB, Martin DMA, et al. Jalview Version 2-A multiple sequence alignment editor and analysis workbench. Bioinformatics. 2009;25(9):1189–1191. 19151095 10.1093/bioinformatics/btp033 PMC2672624

[51] Wright PR, Georg J, Mann M, et al. CopraRNA and IntaRNA: predicting small RNA targets, networks and interaction domains. Nucleic Acids Res. 2014;42:119–123. 10.1093/nar/gku359 PMC4086077 24838564

[52] Wright PR, Richter AS, Papenfort K, et al. Comparative genomics boosts target prediction for bacterial small RNAs. Proc Natl Acad Sci U S A. 2013;110(37):E3487–96. 23980183 10.1073/pnas.1303248110 PMC3773804

[53] Lorenz R, Bernhart SH, Höner Zu Siederdissen C, et al. ViennaRNA Package 2.0. Algorithms Mol Biol. 2011;6(1):26. 22115189 10.1186/1748-7188-6-26 PMC3319429

[54] Newbury SF, Smith NH, Higgins CF. Differential mRNA stability controls relative gene expression within a polycistronic operon. Cell. 1987;51(6):1131–1143. 2446776 10.1016/0092-8674(87)90599-x

[55] Chao Y, Li L, Girodat D, et al. In vivo cleavage map illuminates the central role of RNase E in coding and non-coding RNA pathways. Mol Cell. 2017;65(1):39–51. 28061332 10.1016/j.molcel.2016.11.002 PMC5222698

[56] Thomason MK, Bischler T, Eisenbart SK, et al. Global transcriptional start site mapping using differential RNA sequencing reveals novel antisense RNAs in Escherichia coli. J Bacteriol. 2015;197(1):18–28. 10.1128/JB.02096-14 PMC4288677 25266388

[57] Bossi L, Maloriol D, Figueroa-Bossi N. Porin biogenesis activates the σE response in Salmonella hfq mutants. Biochimie. 2008;90(10):1539–1544. 18585433 10.1016/j.biochi.2008.06.001

[58] De Las Peñas A, Connolly L, Gross CA. The σ(E)-mediated response to extracytoplasmic stress in Escherichia coli is transduced by RseA and RseB, two negative regulators of σ(E). Mol Microbiol. 1997;24(2):373–385. 9159523 10.1046/j.1365-2958.1997.3611718.x

[59] Alba BM, Gross CA. Regulation of the Escherichia coli σE-dependent envelope stress response. Mol Microbiol. 2004;52(3):613–619. 15101969 10.1111/j.1365-2958.2003.03982.x

[60] Rhodius VA, Suh WC, Nonaka G, et al. Conserved and variable functions of the σE stress response in related genomes. PLoS Biol. Internet] 2006; 4:0043–59. [cited 2019 101]. 10.1371/journal.pbio.0040002 PMC1312014 16336047

[61] Udekwu KI, Wagner EGH. Sigma E controls biogenesis of the antisense RNA MicA. Nucleic Acids Res. 2007;35(4):1279–1288. 10.1093/nar/gkl1154 PMC1851643 17267407

[62] Chen S, Zhang A, Blyn LB, et al. MicC, a second small-RNA regulator of omp protein expression in Escherichia coli. J Bacteriol. 2004;186(20):6689–6697. 10.1128/JB.186.20.6689-6697.2004 PMC522180 15466019

[63] Wang X, Xia K, Yang X, et al. Growth strategy of microbes on mixed carbon sources. Nat Commun. 2019;10:1–7. 30894528 10.1038/s41467-019-09261-3 PMC6427025

[64] Li Z, Nimtz M, Rinas U. The metabolic potential of Escherichia coli BL21 in defined and rich medium. Microb Cell Fact Internet] 2014; 13(1):45. 24656150 10.1186/1475-2859-13-45 PMC4021462

[65] Khemici V, Carpousis AJ. The RNA degradosome and poly(A) polymerase of Escherichia coli are required in vivo for the degradation of small mRNA decay intermediates containing REP-stabilizers. Mol Microbiol. Internet] 2004 [cited 2019 1211]; 51(3):777–790. 14731278 10.1046/j.1365-2958.2003.03862.x

[66] Nitzan M, Rehani R, Margalit H. Integration of bacterial small RNAs in regulatory networks. Annu Rev Biophys. 2017;46(1):131–148. 28532217 10.1146/annurev-biophys-070816-034058

[67] Lalaouna D, Carrier M-C, Semsey S, et al. A 3′ external transcribed spacer in a tRNA transcript acts as a sponge for small RNAs to prevent transcriptional noise. Mol Cell. 2015;58(3):393–405. 25891076 10.1016/j.molcel.2015.03.013

[68] Douchin V, Bohn C, Bouloc P. Down-regulation of porins by a small RNA bypasses the essentiality of the regulated intramembrane proteolysis protease RseP in Escherichia coli. J Biol Chem. 2006;281(18):12253–12259. 10.1074/jbc.M600819200 16513633

[69] Yakhnin H, Aichele R, Ades SE, et al. Circuitry linking the global Csr- and σ E -dependent cell envelope stress response systems. J Bacteriol. 2017;199(23). DOI:10.1128/JB.00484-17 PMC5686586 28924029

[70] Bernstein JA, Khodursky AB, Lin P-H-H, et al. Global analysis of mRNA decay and abundance in Escherichia coli at single-gene resolution using two-color fluorescent DNA microarrays. Proc Natl Acad Sci U S A. 2002;99(15):9697–9702. 12119387 10.1073/pnas.112318199 PMC124983

[71] Chang DE, Smalley DJ, Tucker DL, et al. Carbon nutrition of Escherichia coli in the mouse intestine. Proc Natl Acad Sci U S A. 2004;101:7427–7432. 15123798 10.1073/pnas.0307888101 PMC409935

[72] Jones SA, Jorgensen M, Chowdhury FZ, et al. Glycogen and maltose utilization by Escherichia coli O157: h7in the mouse intestine. Infect Immun. 2008;76(6):2531–2540. 18347038 10.1128/IAI.00096-08 PMC2423072

[73] Davis BM, Waldor MK. RNase E-dependent processing stabilizes MicX, a Vibrio cholerae sRNA. Mol Microbiol. 2007;65(2):373–385. 10.1111/j.1365-2958.2007.05796.x PMC1976385 17590231

[74] Fozo EM, Kawano M, Fontaine F, et al. Repression of small toxic protein synthesis by the Sib and OhsC small RNAs. Mol Microbiol. 2008. 10.1111/j.1365-2958.2008.06394.x. PMC2597788 18710431

[75] Fozo EM. New type I toxin-antitoxin families from “wild” and laboratory strains of E. coli. RNA Biol. 2012;9(12):1504–1512. 10.4161/rna.22568 23182878

[76] De RH, Roy A, Danchin A. Analysis of the ptsH-ptsI-crr region in Escherichia coli K-12: nucleotide sequence of the ptsH gene. Gene. 1985. 10.1016/0378-1119(85)90172-6. 2411636

[77] Deutscher J. The mechanisms of carbon catabolite repression in bacteria. Curr Opin Microbiol. 2008;11(2):87–93. 10.1016/j.mib.2008.02.007 18359269

[78] Chen S, Oldham ML, Davidson AL, et al. Carbon catabolite repression of the maltose transporter revealed by X-ray crystallography. Nature. 2013;499(7458):364–368. 23770568 10.1038/nature12232 PMC3875231

