# Supplementary material for: The mRNA derived MalH sRNA contributes to alternative carbon source utilization by tuning maltoporin expression in E. coli
Source: RNA Biol. 2020 Oct 12;18(6):914–31. doi: 10.1080/15476286.2020.1827784 (PMC8081044; doi:10.1080/15476286.2020.1827784)
Supplement: Supplemental Material [file KRNB_A_1827784_SM8936.zip › Supplementary information/Supplementary Figure legends.docx]

**Supplementary Figure legends**

**Supplementary Figure 1.** **Conservation and target prediction analyses of MalH.**

(**A**) MalH contains conserved and variable regions. Sequence conservation analysis of MalH in several Gram-negative bacteria species (Mafft algorithm with defaults); the arrow indicates the 5’-end of MalH; the *malG* stop codon and Rho-independent terminator sites are highlighted; the horizontal black lines indicate the base-pairing regions of MalH with *ptsH* (top), OhsC, *ompA* and *ompC* (bottom) in *E. coli* as predicted by CLASH combined with *in silico* folding (RNACofold). (**B**) MalH is predicted to interact with its targets using two seed regions. Interaction regions within MalH and top target mRNAs predicted by CopraRNA^51,52^. Density plots showing the relative frequency of a specific MalH (Left) or mRNA (Right) nucleotide position in all predicted sRNA-mRNA interactions with a p-value < 0.01 in all considered homologs. The vertical lines indicate local maxima; the aligned regions of the homologs are shown in grey, whereas the interacting regions are shown in arbitrary colors; only the top 20 representative clusters members are shown in the aligned regions, with the gene names indicated on the right. The strains used for the CopraRNA analyses were NC_000913 (*E. coli* MG1655), NC_022912 (*Shigella_dysenteriae*_1617) and NC_018405 (*Enterobacter_cloacae_subsp._cloacae*_ENHKU01).

**Supplementary Figure 2. Conservation of the *ompC* 5’UTR and sRNA binding sites.**

Interaction of *ompC* 5’UTR with various sRNA. (**A**) 5’UTR of *ompC* (from 2312864 to 2312743 in K-12 substr. MG1655) aligned to homologous regions of other enterobacteria. Binding regions of known interacting sRNAs and two transcription start sites (ompCp1 and ompCp2) are indicated. (**B**) Secondary structure as predicted by RNAalifold (http://rna.tbi.univie.ac.at/RNAWebSuite/RNAalifold.cgi) for the alignment in A with Shine-Dalgarno (SD) stem-loop. (**C**) *OmpC* 5’UTR (green) base-paired with MalH (red).

**Supplementary Figure 3.** **The MalH SM mutant is stably expressed.** (**A**) Northern blot analysis of wild-type MalH and seed mutant (MalH SM). Note that because attempts to detect both MalH species with a single oligonucleotide probe to detect both RNA species were not successful, we used two probes that hybridised near the 5’ end of MalH and detected either the wild-typ or mutant allele. After probing for the MalH SM transcript, we re-probed the same blot with a RybB probe, to demonstrate that MalH is indeed expressed at high levels. (**B**) Because we used different probes for detecting MalH RNAs in the Northern blot analyses, we were unable to determine the relative expression levels of the two RNAs. Therefore, to complement these results, we performed qPCR analyses to detect MalH and MalH SM. As a negative control, we included an empty pBAD plasmid, which enabled us to detect endogenous levels of *malG/*MalH. These data show that the levels of the endogenous transcripts are orders of magnitude lower than MalH and MalH SM expressed from the plasmid. Thus, we conclude that the qPCR signal primarily originates from MalH and MalH SM expressed from the pBAD plasmid. These data show that both RNAs are expressed at similar levels relative to the *recA* mRNA transcript.
