# Supplementary material for: The mRNA derived MalH sRNA contributes to alternative carbon source utilization by tuning maltoporin expression in E. coli
Source: RNA Biol. 2020 Oct 12;18(6):914–31. doi: 10.1080/15476286.2020.1827784 (PMC8081044; doi:10.1080/15476286.2020.1827784)
Supplement: Supplemental Material [file KRNB_A_1827784_SM8936.zip › Supplementary information/Supplementary_Figure_4.pdf]

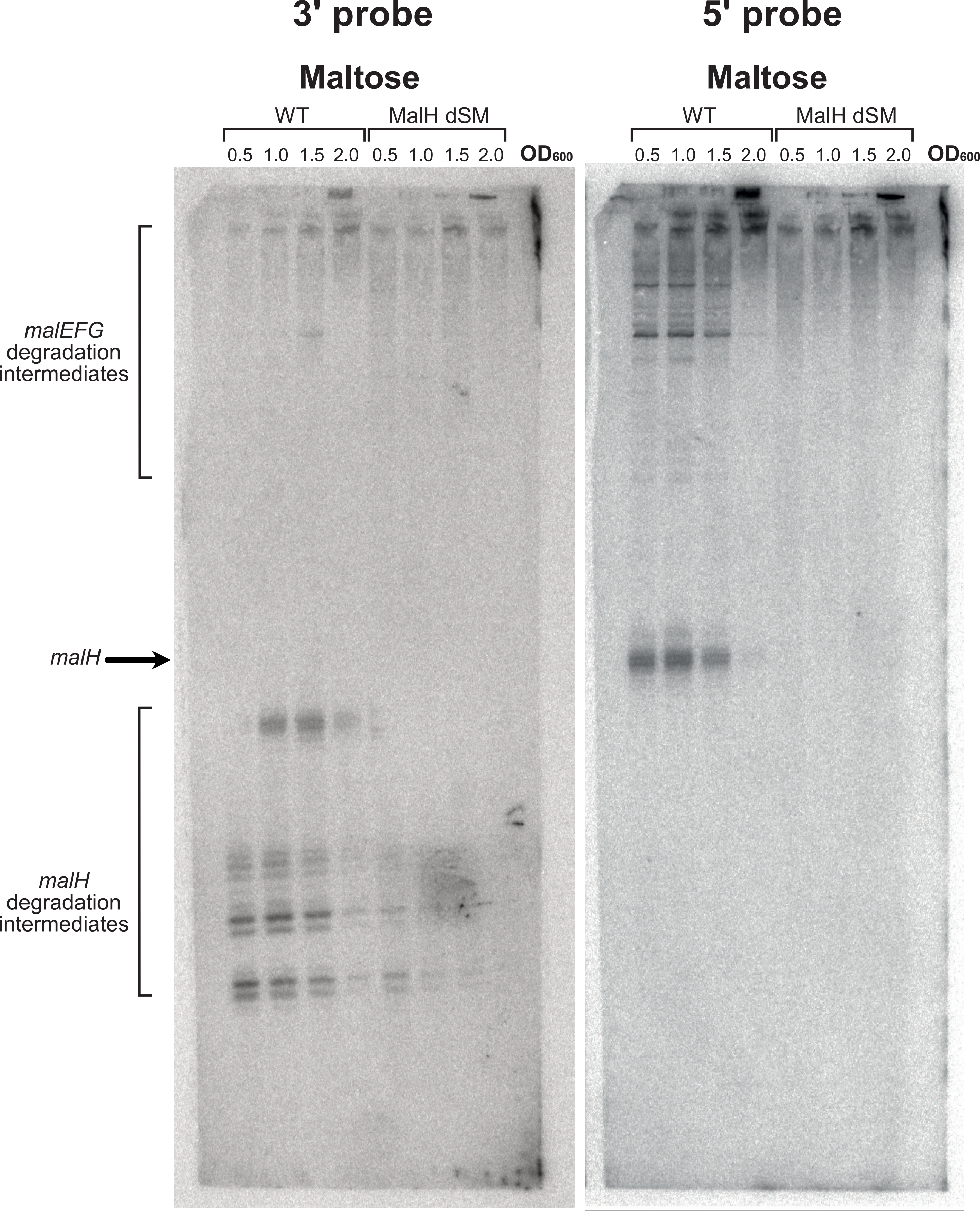
3' probe
Maltose
WT
MalH dSM
0.5 1.0 1.5 2.0 0.5 1.0 1.5 2.0
OD600
malEFG degradation intermediates
malH
malH degradation intermediates
5' probe
Maltose
WT
MalH dSM
0.5 1.0 1.5 2.0 0.5 1.0 1.5 2.0
OD600
