# Supplementary figures and images for: The mRNA derived MalH sRNA contributes to alternative carbon source utilization by tuning maltoporin expression in E. coli
Source: RNA Biol. 2020 Oct 12;18(6):914–31. doi: 10.1080/15476286.2020.1827784 (PMC8081044; doi:10.1080/15476286.2020.1827784)

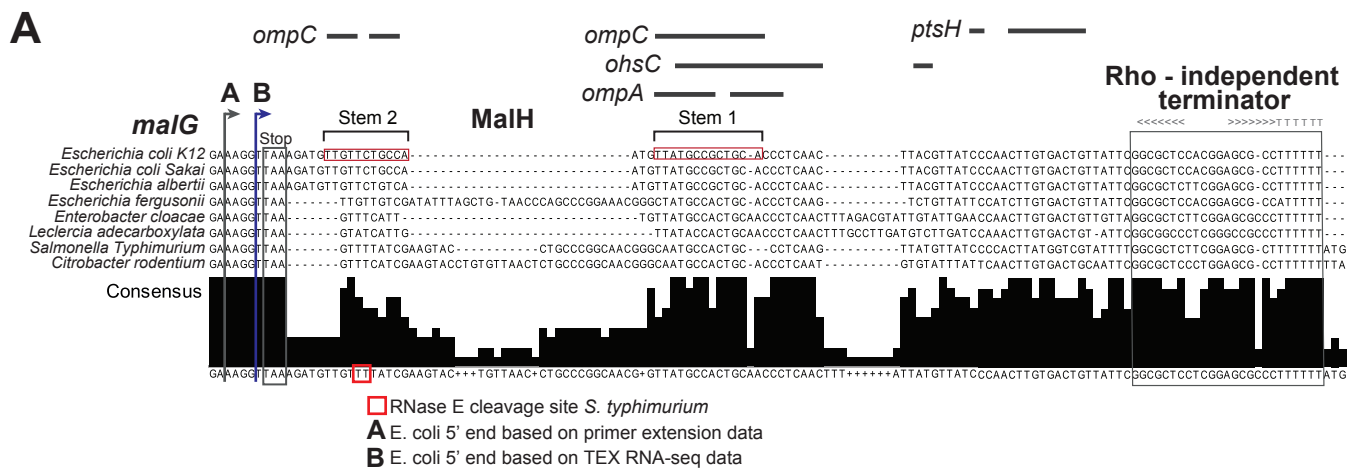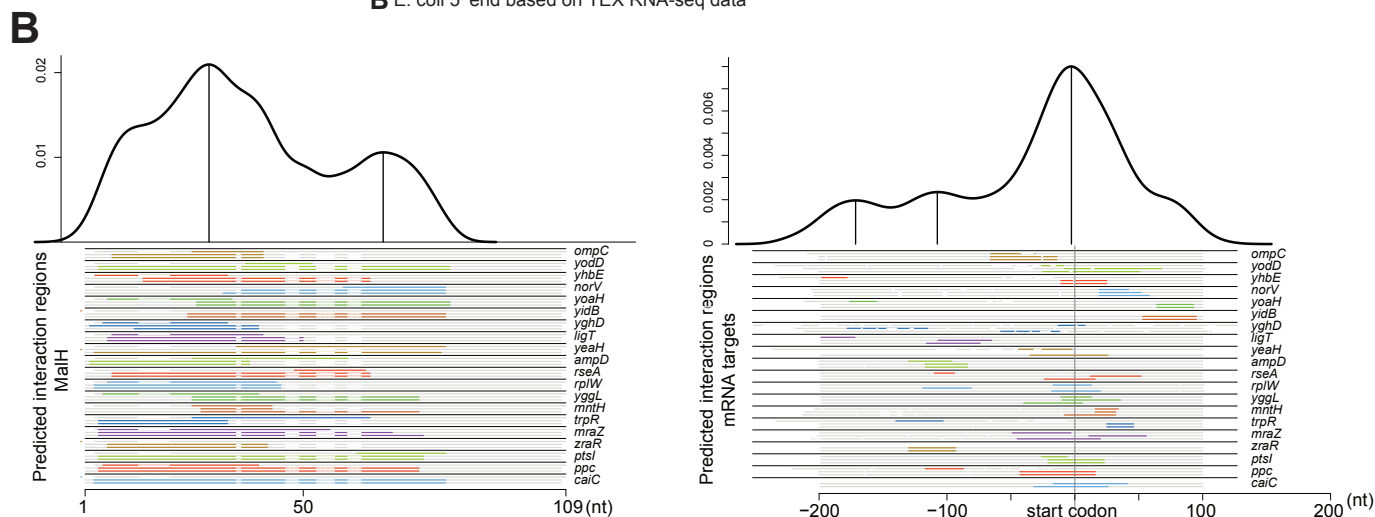

Supplement: Supplemental Material [file KRNB_A_1827784_SM8936.zip › Supplementary information/Supplementary_Figure_1.pdf]

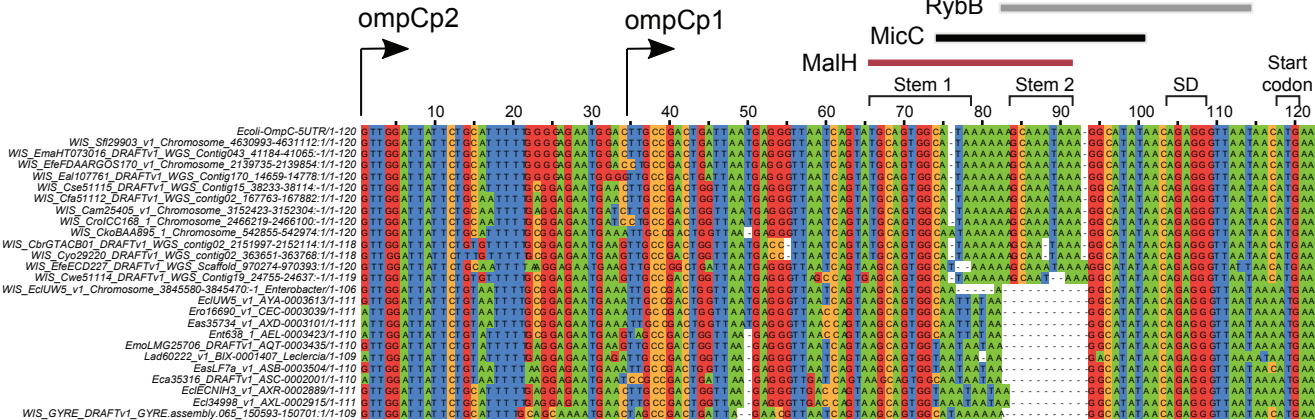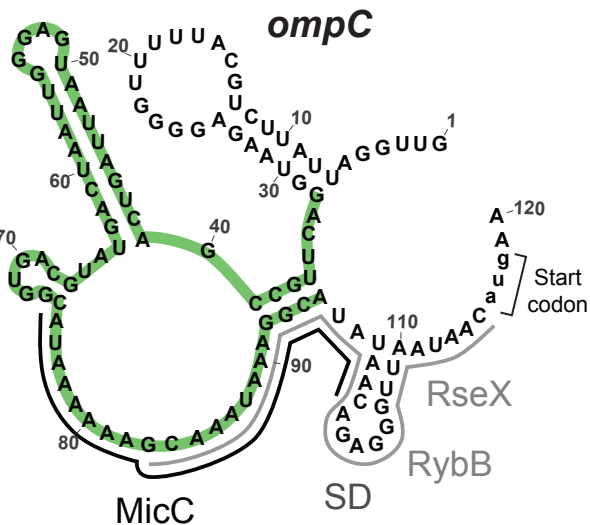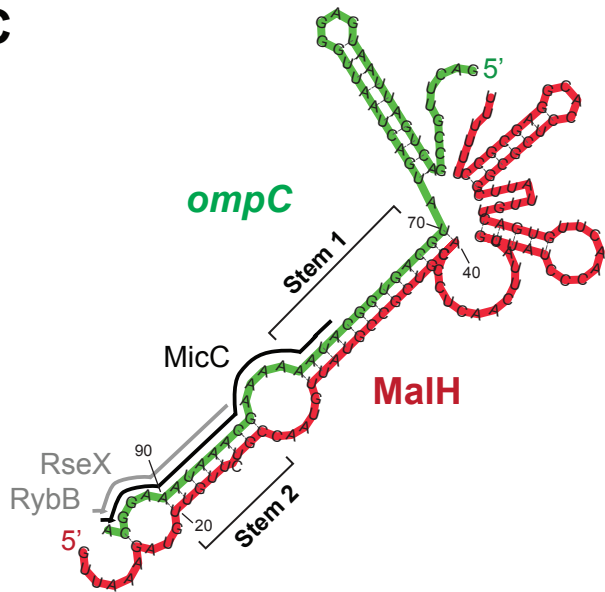

Supplement: Supplemental Material [file KRNB_A_1827784_SM8936.zip › Supplementary information/Supplementary_Figure_2.pdf]

**A**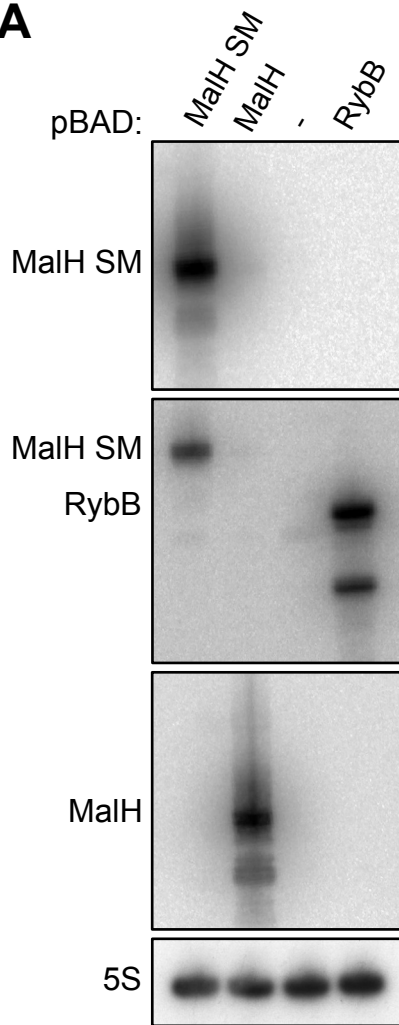**B**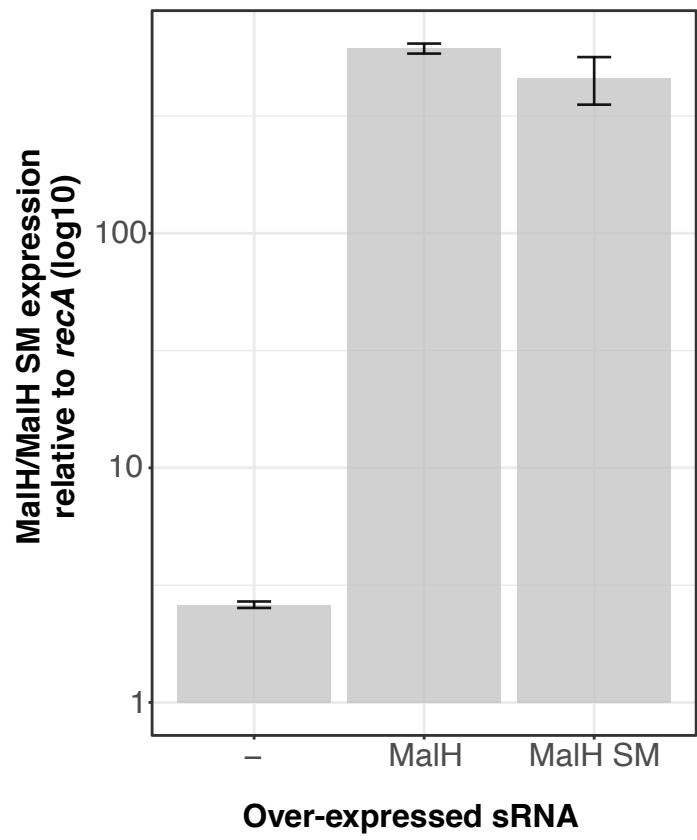

Supplement: Supplemental Material [file KRNB_A_1827784_SM8936.zip › Supplementary information/Supplementary_Figure_3.pdf]
